# Adiponectin receptor agonist AdipoRon ameliorates renal inflammation in diet-induced obese mice and endotoxin-treated human glomeruli ex vivo

**DOI:** 10.1007/s00125-021-05473-9

**Published:** 2021-05-14

**Authors:** Sonja Lindfors, Zydrune Polianskyte-Prause, Rim Bouslama, Eero Lehtonen, Miia Mannerla, Harry Nisen, Jukka Tienari, Hanne Salmenkari, Richard Forsgård, Tuomas Mirtti, Markku Lehto, Per-Henrik Groop, Sanna Lehtonen

**Affiliations:** 1 Research Program for Clinical and Molecular Metabolism, Faculty of Medicine, University of Helsinki, Helsinki, Finland; 2 Department of Pathology, University of Helsinki, Helsinki, Finland; 3 Folkhälsan Institute of Genetics, Folkhälsan Research Center, Helsinki, Finland; 4 Abdominal Center, Nephrology, University of Helsinki and Helsinki University Hospital, Helsinki, Finland; 5 Abdominal Center, Urology, University of Helsinki and Helsinki University Hospital, Helsinki, Finland; 6 Department of Pathology, University of Helsinki and Helsinki University Hospital, Helsinki, Finland; 7 Pharmacology, Faculty of Medicine, University of Helsinki, Helsinki, Finland; 8 Research Program in Systems Oncology, Faculty of Medicine, University of Helsinki, Helsinki, Finland; 9 Department of Diabetes, Central Clinical School, Monash University, Melbourne, VIC Australia

**Keywords:** Adiponectin, AdipoRon, Diabetes, Diabetic kidney disease, Inflammation, Inflammatory cytokines, LPS, Obesity, Obesity-related kidney disease, Podocyte

## Abstract

**Aims/hypothesis:**

Chronic low-grade inflammation with local upregulation of proinflammatory molecules plays a role in the progression of obesity-related renal injury. Reduced serum concentration of anti-inflammatory adiponectin may promote chronic inflammation. Here, we investigated the potential anti-inflammatory and renoprotective effects and mechanisms of action of AdipoRon, an adiponectin receptor agonist.

**Methods:**

Wild-type DBA/2J mice were fed with high-fat diet (HFD) supplemented or not with AdipoRon to model obesity-induced metabolic endotoxaemia and chronic low-grade inflammation and we assessed changes in the glomerular morphology and expression of proinflammatory markers. We also treated human glomeruli ex vivo and human podocytes in vitro with AdipoRon and bacterial lipopolysaccharide (LPS), an endotoxin upregulated in obesity and diabetes, and analysed the secretion of inflammatory cytokines, activation of inflammatory signal transduction pathways, apoptosis and migration.

**Results:**

In HFD-fed mice, AdipoRon attenuated renal inflammation, as demonstrated by reduced expression of glomerular activated NF-κB p65 subunit (NF-κB-p65) (70%, *p* < 0.001), TNFα (48%, *p* < 0.01), IL-1β (51%, *p* < 0.001) and TGFβ (46%, *p* < 0.001), renal IL-6 and IL-4 (21% and 20%, *p* < 0.05), and lowered glomerular F4/80-positive macrophage infiltration (31%, *p* < 0.001). In addition, AdipoRon ameliorated HFD-induced glomerular hypertrophy (12%, *p* < 0.001), fibronectin accumulation (50%, *p* < 0.01) and podocyte loss (12%, *p* < 0.001), and reduced podocyte foot process effacement (15%, *p* < 0.001) and thickening of the glomerular basement membrane (18%, *p* < 0.001). In cultured podocytes, AdipoRon attenuated the LPS-induced activation of the central inflammatory signalling pathways NF-κB-p65, c-Jun N-terminal kinase (JNK) and p38 mitogen-activated protein kinase (p38-MAPK) (30%, 36% and 22%, respectively, *p* < 0.001), reduced the secretion of TNFα (32%, *p* < 0.01), and protected against podocyte apoptosis and migration. In human glomeruli ex vivo, AdipoRon reduced the LPS-induced secretion of inflammatory cytokines IL-1β, IL-18, IL-6 and IL-10.

**Conclusions/interpretation:**

AdipoRon attenuated the renal expression of proinflammatory cytokines in HFD-fed mice and LPS-stimulated human glomeruli, which apparently contributed to the amelioration of glomerular inflammation and injury. Mechanistically, based on assays on cultured podocytes, AdipoRon reduced LPS-induced activation of the NF-κB-p65, JNK and p38-MAPK pathways, thereby impelling the decrease in apoptosis, migration and secretion of TNFα. We conclude that the activation of the adiponectin receptor by AdipoRon is a potent strategy to attenuate endotoxaemia-associated renal inflammation.

**Graphical abstract:**

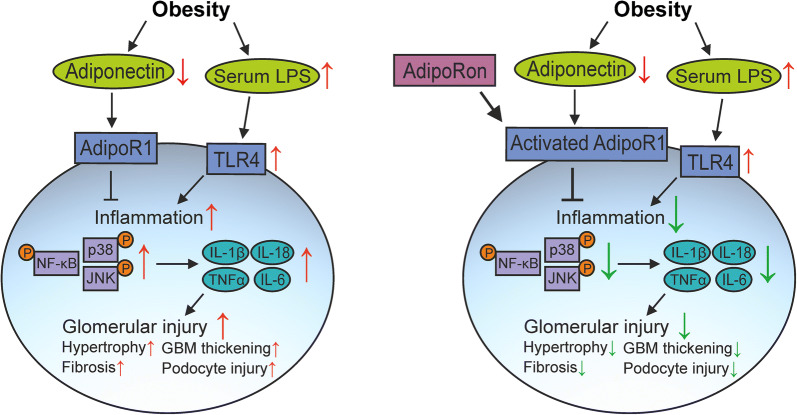

**Supplementary Information:**

The online version contains peer-reviewed but unedited supplementary material available at 10.1007/s00125-021-05473-9.

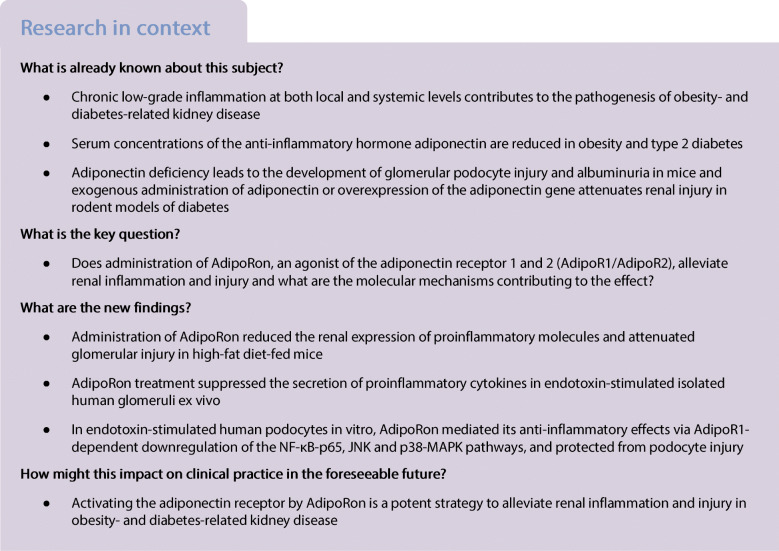



## Introduction

Obesity and diabetes are important risk factors for the development of chronic kidney disease [1]. Both conditions are characterised by increased serum levels of lipopolysaccharides (LPSs), a state recognised as metabolic endotoxaemia, and chronic low-grade inflammation [2–5], and thus share a common trigger for the progression of renal injury. In response to metabolic stress and inflammatory milieu, renal cells secrete proinflammatory molecules, which activate an innate immunity response and promote renal abnormalities [4, 5]. Injury to podocytes, the specialised glomerular epithelial cells that maintain the glomerular filtration barrier, is considered a major contributor to both obesity- and diabetes-related kidney disease [1, 6].

Obesity and type 2 diabetes are associated with decreased serum concentrations of adiponectin, a peptide hormone secreted by the adipose tissue [7]. As adiponectin has anti-inflammatory properties [7], its deficiency may promote chronic inflammation. Incidentally, adiponectin-deficient mice develop podocyte injury and albuminuria even in the absence of diabetes [8], and renal injury is further aggravated by experimental diabetes [8, 9]. Correspondingly, exogenous administration of adiponectin or overexpression of the adiponectin gene attenuates renal injury in rodent models of diabetes [10, 11]. Both in obese individuals without diabetes and in individuals with type 2 diabetes, the serum adiponectin concentrations inversely correlate with the degree of albuminuria [12–14].

In the present study, we hypothesised that enhancing anti-inflammatory signalling by activating the adiponectin signalling pathway with AdipoRon, an orally active small-molecule agonist of adiponectin receptor 1/2 (AdipoR1/2) [15], prevents inflammation-related renal injury. We investigated whether AdipoRon ameliorates renal inflammation and injury using high-fat diet-fed obese mice as a model of metabolic endotoxaemia and local tissue inflammation [2]. In addition, to characterise the molecular mechanisms underlying the anti-inflammatory effects of AdipoRon, we used endotoxin-stimulated human glomeruli ex vivo and human podocytes in vitro as model systems.

## Methods

### Reagents

AdipoRon was purchased from Enamine (#Z32352521; Kiev, Ukraine) for the mouse experiment and from MilliporeSigma (#SML0998) for the cell culture studies. Antibodies (electronic supplementary material [ESM] Table 1) were validated by knockout/overexpression experiments or passed the manufacturer’s application-specific testing standards. Further reagent details are given in the ESM Methods.

### Animals

Five-week-old male DBA/2J mice were purchased from Charles River Laboratories (Lyon, France). After acclimation for 1 week, mice were randomly assigned to experimental groups (2 mice/cage) based on body weight, and fed with either low-fat diet (LFD; 10% kJ fat, #D12450B, Research Diets, NJ, USA) (*n* = 8), high-fat diet (HFD; 60% kJ fat, #D12492, Research Diets) (*n* = 14), or HFD supplemented with AdipoRon (380 mg/kg diet, custom-made by Research Diets) (*n* = 14), for 9 weeks. The dose was chosen based on previous studies administering AdipoRon 30–50 mg/kg body weight [15, 16], yielding a mean dose of AdipoRon 51.5 mg/kg at the beginning and 28.2 mg/kg at the end of the experiment, as the mice gained weight. The experiments were performed according to the Federation of European Laboratory Animal Science Associations (FELASA) guidelines (2015) and approved by the National Animal Experiment Board.

Body weight and food intake were monitored weekly. After 7 weeks of treatment, blood glucose was measured after 6 h fasting. Urine and faecal pellets were collected individually in metabolic cages (24 h) at the baseline and after 8 weeks of treatment. A detailed description of the housing and experimental procedures is given in ESM Methods.

### Harvesting of organs

After 9 weeks of treatment, mice were killed for tissue collection. Serum samples were prepared from blood obtained by cardiac puncture. Renal cortex tissue was either snap-frozen, or fixed in 10% (vol./vol.) formalin followed by paraffin embedding, or embedded and frozen in optimal cutting temperature (OCT) compound or fixed in 1.5% (vol./vol.) glutaraldehyde, 3% (wt/vol.) paraformaldehyde, 5% (wt/vol.) sucrose in 0.1 mol/l phosphate buffer (pH 7.4) and processed for electron microscopy as described [17]. Intestinal tissue was snap-frozen. Preparation of protein lysates from renal tissue extracts is described in ESM Methods.

### Serum and urine biochemical analysis

Serum concentrations of adiponectin, insulin and LPS were measured by ELISA kits (see ESM Methods for details). The urinary albumin and creatinine concentrations were measured at the Biochemical Analysis Core for Experimental Research (University of Helsinki).

### Immunoblotting

Expression of AdipoR1, toll-like receptor 4 (TLR4), cleaved caspase-3 and the phosphorylated forms of NF-kappa-B inhibitor alpha (IκBα), NF-κB-p65, c-Jun N-terminal kinase (JNK) and p38 mitogen-activated protein kinase (p38-MAPK) was studied by immunoblotting. Equal amounts of protein samples prepared from tissue or cellular extracts were separated by SDS-PAGE, transferred to PVDF-FL membranes (Millipore, Billerica, MA, USA), blocked with Odyssey blocking buffer (LI-COR, Lincoln, NE, USA) diluted 1:1 with TRIS-buffered saline and incubated with primary and secondary antibodies (for antibody details, see ESM Table 1). Membranes were analysed by Odyssey Infrared Imaging System and Image Studio software (LI-COR). See ESM Methods for details.

### Iohexol experiment for intestinal permeability

After 6 weeks of treatment, eight mice randomly selected from each group were tested for intestinal permeability by the iohexol assay. Briefly, mice were administered 4 ml/kg iohexol by gavage and placed into metabolic cages for urine collection (24 h). Urinary iohexol concentration was measured by ELISA. See ESM Methods for details.

### Faecal albumin assay

Albumin concentration in faecal extractions prepared from faecal pellets collected after 8 weeks of treatment was measured by ELISA. See ESM Methods for details.

### Intestinal alkaline phosphatase assay

The intraluminal contents of caecum and distal colon were homogenised in extraction buffer. Intestinal alkaline phosphatase (IAP) activity was determined by p-nitrophenyl phosphate (pNPP) assay. See ESM Methods for details.

### Immunohistochemistry and morphometric analysis

Four-micrometre-thick sections were prepared for immunohistochemistry or periodic acid–Schiff (PAS) staining using standard procedures. After deparaffinisation and antigen retrieval, sections were incubated with antibodies against TGFβ, TNFα, IL-1β, phospho-NF-κB-p65, F4/80, fibronectin and Wilms’ tumour protein (WT1). For details on antigen retrieval conditions and antibodies, see ESM Methods and ESM Table 1, respectively. Normal rabbit or mouse IgGs were used as negative controls. Detection was performed using Brightvision Poly-HRP-anti-Rabbit IgG kit (for mouse tissue; ImmunoLogic, Amsterdam, the Netherlands), EnVision+ immunohistochemistry kit (for mouse tissue; Dako, Santa Clara, USA) or EnVision FLEX+ immunohistochemistry kit (for human tissue; Dako) and 3,3′-diaminobenzidine (Dako). Slides were counterstained with Mayer’s Hematoxylin (Dako). Images were obtained using 3DHISTECH Pannoramic 250 FLASH II digital slide scanner (Genome Biology Unit supported by HiLIFE and the Faculty of Medicine, University of Helsinki, and Biocenter Finland) at 20× magnification. Glomerular staining area and total glomerular tuft area in PAS-stained sections was quantified from 15–20 glomeruli/section using the Histoquant module of the QuantCenter software version 2.0 (3DHISTECH, Budapest, Hungary). For podocyte count, WT1-positive nuclei were counted from glomeruli larger than 70 μm diameter to ensure sectioning through the centre of the glomerulus.

### Cytokine measurements

Cytokines were measured from renal cortex lysates (IL-4, IL-6) and culture media supernatants of podocytes or isolated human glomeruli (IL-1β, IL-6, IL-8, IL-10, IL-18, TNFα) with multiplex Q-Plex cytokine assays (Quansys Biosciences, Logan, UT, USA). For details, see ESM Methods.

### Macrophage polarisation analysis

Double immunofluorescence staining was performed on OCT-embedded frozen renal cortical sections (5 μm) against CD16/32 (M1 marker) or CD206 (M2 marker) and F4/80 (total macrophage marker). Images were generated using 3DHISTECH Pannoramic 250 FLASH II digital slide scanner at 40× magnification. See ESM Methods for details on staining procedure and image analysis.

### Electron microscopy

Transmission electron microscopy images were obtained and analysed as described [17] (*n* = 3 mice for each group). Mean podocyte foot process width was analysed from 5–7 glomeruli/mouse and the thickness of glomerular basement membrane (GBM) from three glomeruli/mouse. See ESM Methods for details.

### Cell culture and treatments

Conditionally immortalised human podocytes AB8/13 [18] (provided by M. Saleem [Southmead Hospital, University of Bristol, Bristol, UK]) were maintained at 33°C (5% CO_2_) in RPMI-1640 (2 g/l glucose) supplemented with 10% (vol./vol.) FBS, 100 U/ml penicillin, 100 μg/ml streptomycin, 2 mmol/l glutamine and 1% (vol./vol.) insulin-transferrin-selenium (ITS). Podocytes were differentiated at 37°C for 12–14 days, pre-treated with 12 μmol/l AdipoRon (maximum serum concentration in AdipoRon-administered mice [15]) or DMSO for 2 h, with subsequent stimulation with 200 ng/ml LPS for 1 h (phospho-proteins), 24 h (migration, cytokines) or 48 h (apoptosis). Cells were tested negative for mycoplasma contamination. Preparation of protein lysates from cell extracts is described in ESM Methods.

### Generation of stable AdipoR1 knockdown podocyte line

Stable AdipoR1 knockdown human podocyte cell line was generated by lentiviral delivery of human *ADIPOR1* short hairpin RNA (shRNA) into proliferating AB8/13 podocytes. Infected podocytes were selected with puromycin for 9 days. See ESM Methods for details.

### Migration assay

Podocyte migration was measured by in vitro scratch assay as described [19]. Briefly, a scratch was made on a cell monolayer, and the plate was imaged at 0 h and after 24 h of treatment with Cytation5 Multi-Mode Reader (BioTek, Winooski, VT, USA). The number of migrated cells was counted from captured images. See ESM Methods for details.

### Apoptosis assay

Podocyte apoptosis was measured by double staining for annexinV-FITC and 7-aminoactinomycin D (7-AAD) (BD Biosciences, Franklin Lakes, NJ, USA) as described [20]. Cells positive for annexinV-FITC but negative for 7-AAD were defined as apoptotic. See ESM Methods for details.

### Isolation of human glomeruli

Use of human material was approved by the Ethical Committee of the Hospital District of Helsinki and Uusimaa. Written informed consent was obtained from all study participants. Renal cortex tissue was collected from the non-malignant part of the nephrectomies performed at the Helsinki University Hospital. Characteristics of the participants (*n* = 9) are described in ESM Table 2. Glomeruli were isolated by graded sieving with 425/250/150 μm sieves (Retsch, Haan, Germany). Immediately after isolation, glomeruli were evenly divided to culture plates (*n* = 2–3 wells/treatment, 500 μl/well) in DMEM (1.0 g/l glucose) supplemented with 15% (vol./vol.) FBS, 100 U/ml penicillin, 100 μg/ml streptomycin and 2 mmol/l glutamine, stimulated or not with 200 ng/ml LPS and 12 μmol/l AdipoRon or DMSO, and incubated in a 37°C 5% CO_2_ humidified incubator for 24 h. After collecting culture media, glomeruli were sonicated in 100 mmol/l PBS pH 7.4 supplemented with 0.5 mol/l potassium iodide and proteinase/phosphatase inhibitors, and lysed with 1% (vol./vol.) Triton X-100, 1% (vol./vol.) Igepal and 0.1% (wt/vol.) SDS. The concentration of secreted cytokines (pg/ml) was normalised to the total protein content of corresponding glomerular lysate (μg).

### Statistics

Statistical analyses were performed using SPSS version 25.0 (IBM, Chicago, IL, USA) and data presented as mean ± SD unless otherwise specified. To compare differences between groups, unpaired two-tailed Student’s *t* test (two groups), one-way ANOVA or Kruskal–Wallis test with Bonferroni or Tamhane post hoc test was performed (see ESM Methods for details) unless otherwise specified. Mouse weight gain was analysed by two-way repeated measures ANOVA using GraphPad Prism software version 9.0 (San Diego, CA, USA). The differences in cytokine secretion (absolute concentration [pg/ml]/total protein [μg]) between the treatment groups of human glomeruli were analysed using Friedman test for three-group comparison with Bonferroni-adjusted post hoc Wilcoxon signed-rank test. The relative effect of LPS-AdipoRon treatment in comparison with corresponding LPS control (set to 100%) was evaluated using one-sample Wilcoxon signed-rank test (against 100%). Bivariate correlations were analysed with Pearson’s test. A *p* value <0.05 was considered statistically significant. Details on randomisation, blinding, exclusion criteria and sample replicates are given in ESM Methods.

## Results

### AdipoRon reduces weight gain in HFD-fed mice

**Fig. 1:**
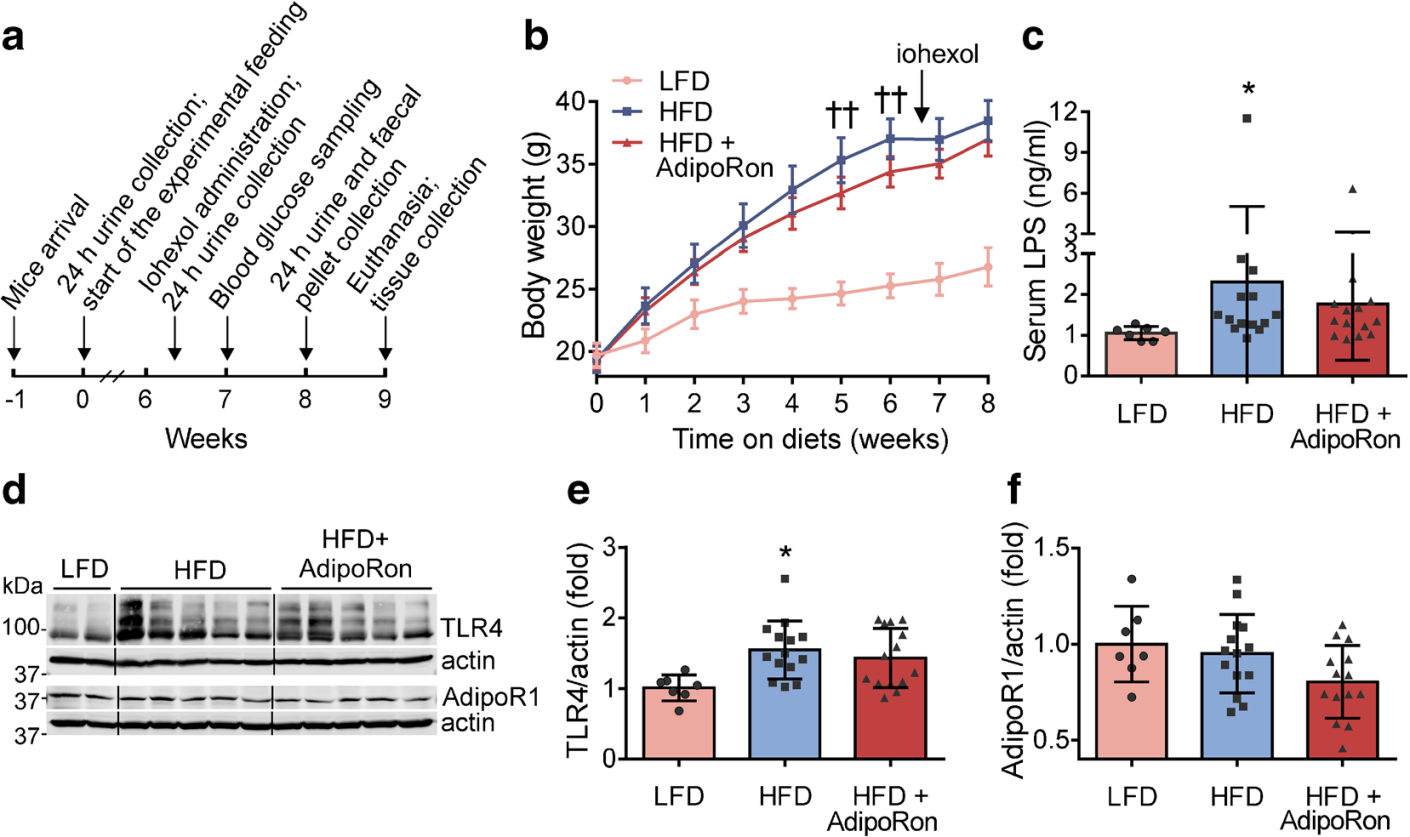
Administration of AdipoRon reduces weight gain without affecting the markers of metabolic endotoxaemia in HFD-fed mice. (**a**) Schematic outline of the study design. (**b**) Body weights of the mice determined at 1 week intervals. The iohexol experiment for intestinal permeability was performed at 6.5 weeks on HFD. Data are presented as mean ± 95% CI and assessed by two-way repeated measures ANOVA with a subsequent Bonferroni post hoc test. LFD, *n* = 7–8; HFD, *n* = 14; HFD-AdipoRon, *n* = 14. (**c**) Serum concentrations of LPS at 9 weeks of treatment. Data were assessed by Kruskal–Wallis test, due to non-normal distribution, with a subsequent Bonferroni post hoc test. LFD, *n* = 7; HFD, *n* = 14; HFD-AdipoRon, *n* = 14. (**d**) Representative immunoblots for TLR4 and AdipoR1 in mouse renal cortical lysates. (**e**, **f**) The expression levels of TLR4 (**e**) and AdipoR1 (**f**) normalised to actin quantified from each mouse as in (**d**). Data were assessed by one-way ANOVA with a subsequent Bonferroni post hoc test. LFD, *n* = 7; HFD, *n* = 14; HFD-AdipoRon, *n* = 14. *p < 0.05 vs LFD. ^††^*p* < 0.01 HFD-AdipoRon vs HFD

Obesity was induced by HFD feeding for 9 weeks in mice (Fig. 1a,b). AdipoRon treatment reduced the HFD-induced body weight by 7% (*p* < 0.01) (Fig. 1b) without affecting food intake (ESM Fig. 1). HFD-fed mice showed characteristics of metabolic endotoxaemia, including elevated serum LPS concentration (Fig. 1c) and renal cortical expression of TLR4, the receptor for LPS, in comparison with LFD-fed mice (Fig. 1d,e), whereas AdipoRon-treated mice did not differ from mice on LFD or HFD (Fig. 1c–e). Serum adiponectin concentration was lowered in HFD- and HFD-AdipoRon-fed mice in comparison with LFD-fed mice (ESM Table 3), whereas renal expression of AdipoR1, the predominantly expressed form of the adiponectin receptors in the kidney [8], did not differ between the groups (Fig. 1d,f). Blood glucose and serum insulin concentrations were elevated by HFD but were not reduced by AdipoRon (ESM Table 3).

We also assessed HFD-induced intestinal permeability, which contributes to increased translocation of microbial endotoxins into the circulation [21] and may thus predispose to renal injury [22]. The level of urinary iohexol, representing iohexol permeated across the intestinal wall to the circulation [23], did not differ between the groups 24 h after oral administration of iohexol (ESM Fig. 2a), indicating no change in the intestinal permeability. The faecal albumin level, another indicator of intestinal leakage [23], did not differ between the groups either (ESM Fig. 2b). As the serum LPS concentration was elevated in the HFD-fed group (Fig. 1c), we examined the intraluminal activity of the IAP, which detoxifies bacterial LPS [21]. The IAP activity was increased by HFD in the caecum and distal colon and was not affected by AdipoRon (ESM Fig. 2c,d).

Unlike HFD-AdipoRon-treated mice, HFD-fed mice suffered from a transient weight loss after iohexol administration, possibly resulting from a transitory iohexol-induced diarrhoea (ESM Fig. 2e), as demonstrated by post- to pre-treatment ratio for body weight (ESM Fig. 2f). As a result, the weight difference between HFD- and HFD-AdipoRon-fed mice was lost (Fig. 1b).

### AdipoRon ameliorates glomerular structural changes, podocyte loss and fibrosis in HFD-fed mice

**Fig. 2:**
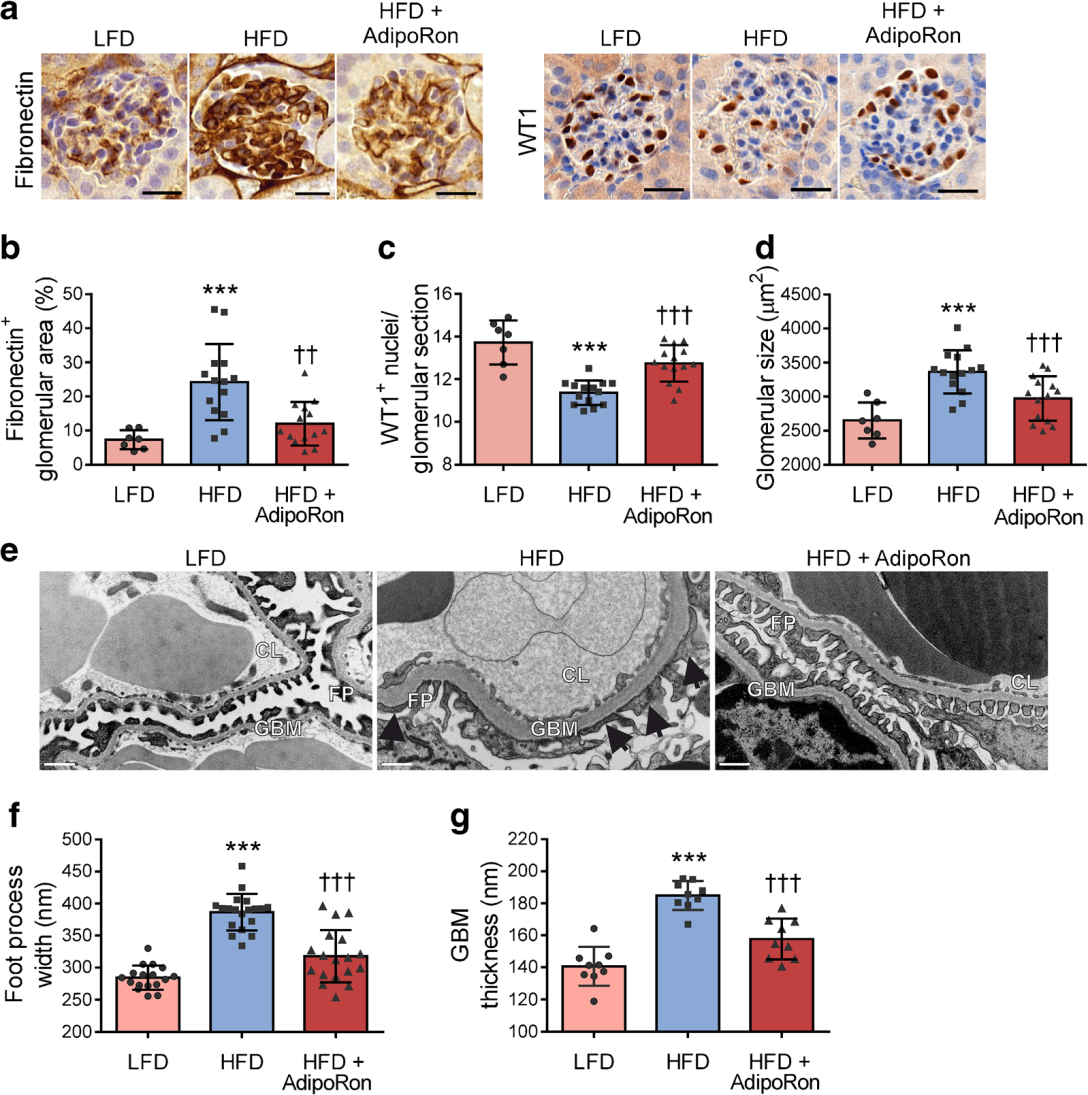
Administration of AdipoRon ameliorates HFD-induced glomerular structural changes, podocyte loss and fibrosis in mice. (**a**) Representative images of immunohistochemical stainings of renal sections for fibronectin and WT1. Scale bar, 25 μm. (**b**, **c**) Quantification of glomerular fibronectin-positive area (**b**) and the number of WT1-positive nuclei per glomerular cross-section (**c**) from 15 glomeruli/mouse. (**d**) Glomerular cross-sectional area of 15 glomeruli/mouse determined from PAS-stained kidney sections. (**b**–**d**) Data were assessed by one-way ANOVA with a subsequent Bonferroni post hoc test. LFD, *n* = 7; HFD, *n* = 14; HFD-AdipoRon, *n* = 14. (**e**) Representative transmission electron microscopy images of glomerular ultrastructure of LFD-fed (*n* = 3), HFD-fed (*n* = 3) and HFD-AdipoRon-fed mice (*n* = 3). Scale bar, 500 nm; original magnification, 30,000×. Arrows indicate effaced foot processes. (**f**) The foot process width of podocytes determined from 5–7 glomeruli/mouse. (**g**) The thickness of GBM determined from three glomeruli/mouse. (**f**, **g**) Scatter dots represent the mean value of each analysed glomerulus. Data were assessed by one-way ANOVA with a subsequent Bonferroni post hoc test. The differences between the groups are also statistically significant (*p* < 0.05) for the mean values of each mouse. ***p < 0.001 vs LFD. ^††^*p* < 0.01 and ^†††^*p* < 0.001 vs HFD. FP, foot process; CL, capillary loop

At 8 weeks of HFD, both control and AdipoRon-treated mice showed a mild increase in 24 h urine albumin excretion in comparison with LFD-fed mice (with no difference in 24 h urine volume between groups) whereas urine albumin/creatinine ratio was not increased by HFD (ESM Table 3). HFD feeding increased glomerular fibronectin expression and glomerular size, indicative of glomerular hypertrophy, and these variables were lowered by AdipoRon by 50% (*p* < 0.01) and 12% (*p* < 0.001), respectively (Fig. 2 a,b,d). Similarly, the HFD-induced reduction in the number of WT1-positive nuclei, indicative of lowered podocyte count, was restored by 12% (*p* < 0.001) by AdipoRon (Fig. 2a,c). AdipoRon also attenuated the HFD-induced widening of the podocyte foot processes by 15% (*p* < 0.001) (Fig. 2e,f) and thickening of the GBM by 18% (*p* < 0.001) (Fig. 2e,g).

### AdipoRon attenuates renal inflammation in HFD-fed mice

**Fig. 3:**
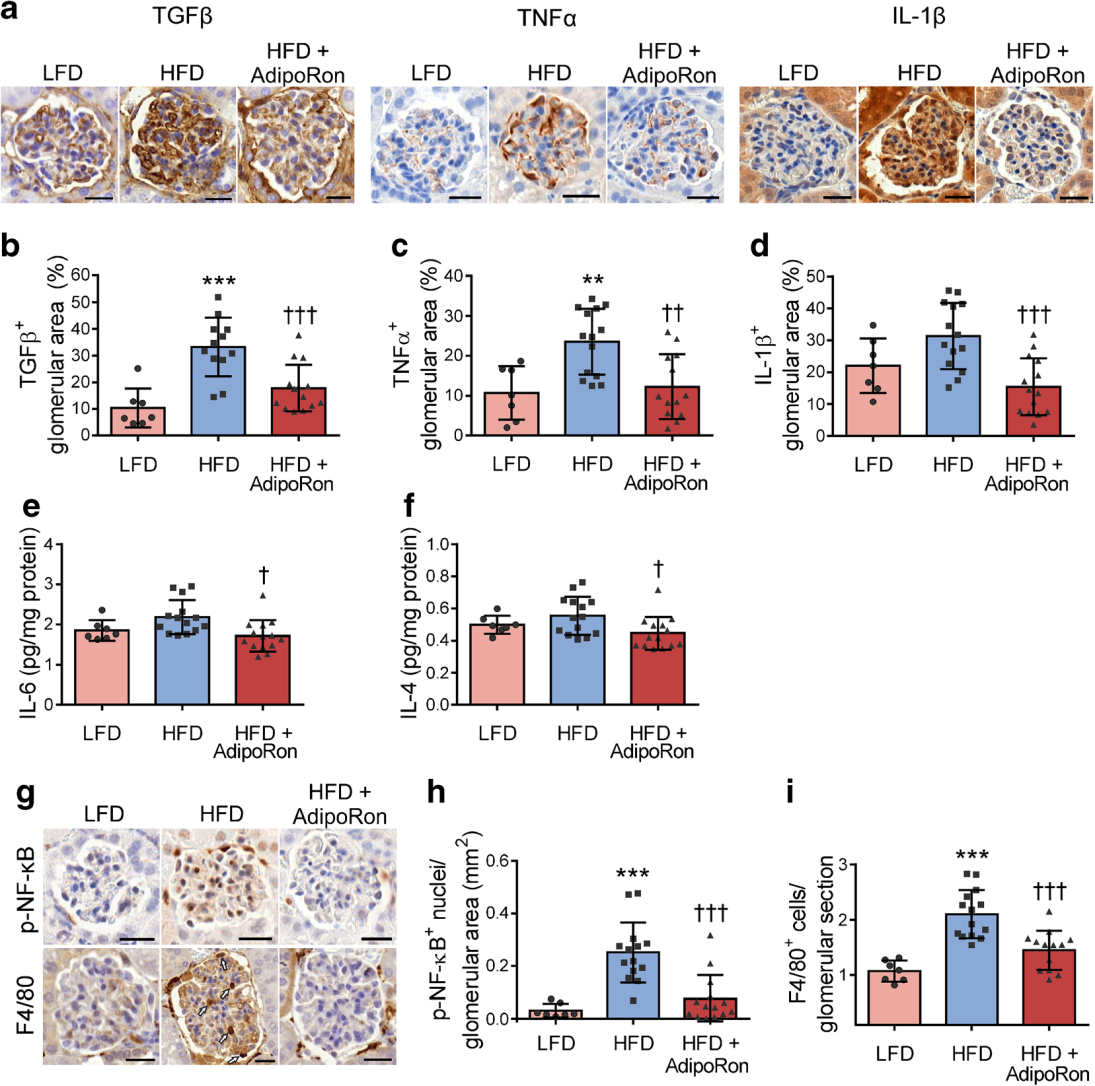
Administration of AdipoRon reduces HFD-induced renal inflammation in mice. (**a**) Representative images of immunohistochemical stainings of renal sections for TGFβ, TNFα and IL-1β. Scale bar, 25 μm. (**b**–**d**) Quantification of glomerular TGFβ-positive area (**b**), TNFα-positive area (**c**) and IL-1β-positive area (**d**) per glomerular cross-section from 15 glomeruli per mouse. LFD, *n* = 7; HFD, *n* = 12–14; HFD-AdipoRon, *n* = 13–14. (**e**, **f**) Concentrations of IL-6 (**e**) and IL-4 (**f**) in renal cortical lysates measured by ELISA. LFD, *n* = 7; HFD, *n* = 14; HFD-AdipoRon, *n* = 14. (**g**) Representative images of immunohistochemical stainings of renal sections for phospho-NF-κB-p65 and F4/80. Scale bar, 25 μm. Arrows indicate F4/80-positive cells. (**h**, **i**) Quantification of glomerular phospho-NF-κB-p65-positive nuclei (**h**) and number of F4/80-positive cells (**i**) per glomerular cross-section from 15 glomeruli per mouse. LFD, *n* = 7; HFD, *n* = 14; HFD-AdipoRon, *n* = 13–14. Data throughout were assessed by one-way ANOVA with a subsequent Bonferroni post hoc test. **p < 0.01 and ***p < 0.001 vs LFD. ^†^*p* < 0.05, ^††^*p* < 0.01 and ^†††^*p* < 0.001 vs HFD

HFD-AdipoRon-treated mice showed reduced glomerular expression of inflammatory cytokines TGFβ (46%, *p* < 0.001) (Fig. 3a,b), TNFα (48%, *p* < 0.01) (Fig. 3a,c) and IL-1β (51%, *p* < 0.001) (Fig. 3a,d) and reduced renal levels of IL-6 and IL-4 (21% and 20%, respectively, *p* < 0.05) (Fig. 3e,f) in comparison with HFD-fed mice. In addition, AdipoRon attenuated the HFD-induced glomerular expression of phosphorylated NF-κB p65 subunit (NF-κB-p65) by 70% (*p* < 0.001) (Fig. 3g,h) and infiltration of F4/80^+^ macrophages by 31% (*p* < 0.001) (Fig. 3g,i). However, the percentage of CD16/CD32^+^ F4/80^+^ cells (M1 macrophages) and the percentage of CD206^+^ F4/80^+^ cells (M2 macrophages) (ESM Fig. 3) did not differ between the groups, indicating that altered macrophage polarisation between the M1 and M2 phenotypes is not involved in this model of renal inflammation.

### AdipoRon reduces LPS-induced inflammatory signalling in cultured podocytes

**Fig. 4:**
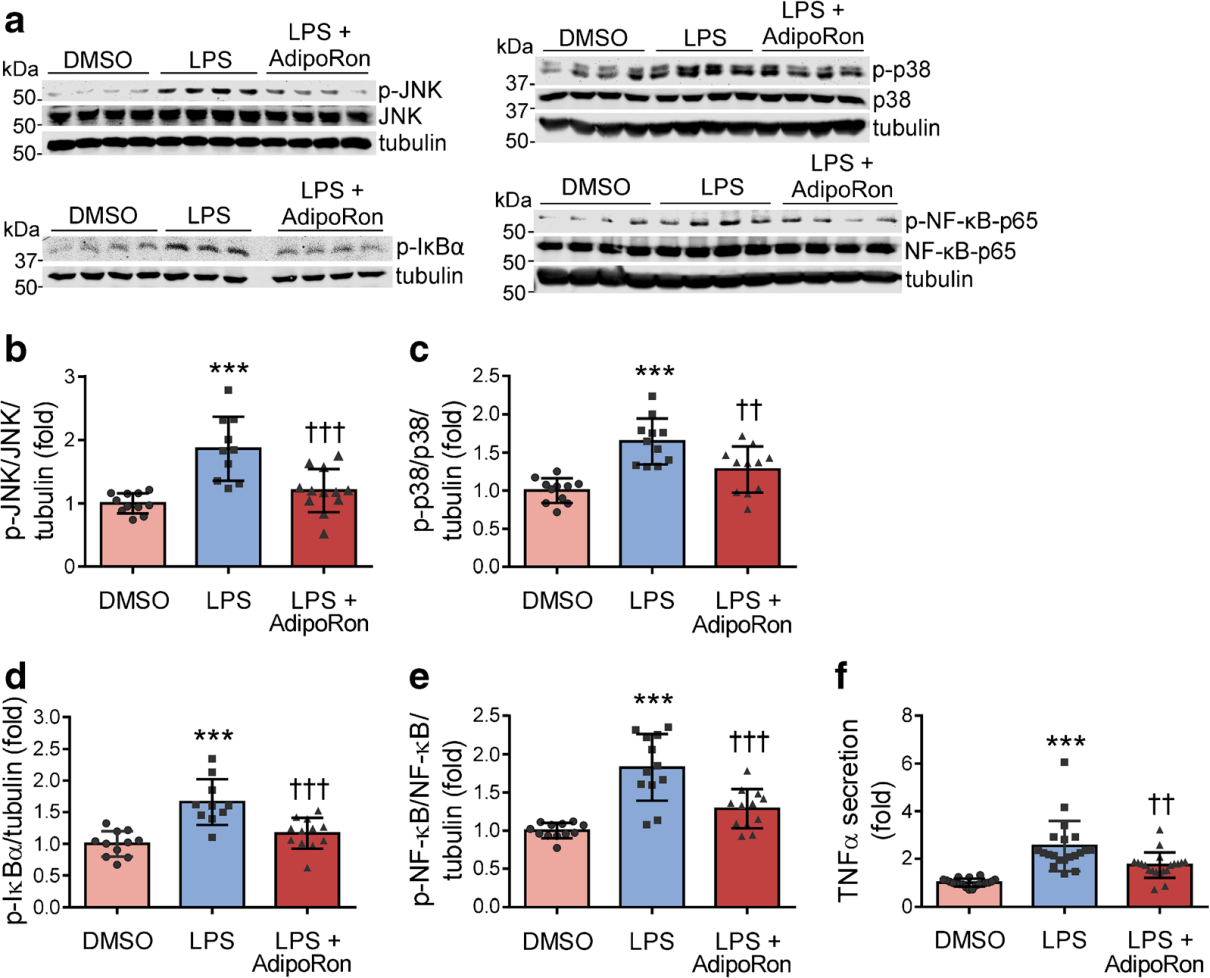
AdipoRon reduces LPS-induced inflammatory signalling in cultured human podocytes. (**a**) Representative immunoblots of lysates prepared from human podocytes treated with AdipoRon or DMSO and stimulated with LPS for 1 h. (**b**–**e**) The expression levels of phospho-JNK (p54 isoform)/JNK (**b**), phospho-p38-MAPK/p38-MAPK (**c**), phospho-IκBα (**d**) and phospho-NF-κB-p65/NF-κB-p65 (**e**) normalised to tubulin quantified from at least three independent experiments as in (**a**). (**f**) The level of TNFα secreted into the cell culture media in response to 24 h LPS stimulation is representative of four independent experiments. Results are normalised to DMSO-treated control. Data throughout were assessed by one-way ANOVA with a subsequent Bonferroni post hoc test. ***p < 0.001 vs DMSO. ^††^*p* < 0.01 and ^†††^*p* < 0.001 vs LPS

To understand the underlying molecular mechanisms through which AdipoRon ameliorates glomerular inflammation, we carried out studies on LPS-stimulated cultured human podocytes. AdipoRon reduced the LPS-induced phosphorylation of IκBα, NF-κB-p65, JNK and p38-MAPK by 30%, 30%, 36% and 22%, respectively (*p* < 0.001) (Fig. 4a–e), indicating that its anti-inflammatory actions are mediated via suppression of the NF-κB-p65, JNK and p38-MAPK pathways. Accordingly, AdipoRon reduced the LPS-induced secretion of TNFα by 32% (*p* < 0.01) (Fig. 4f). AdipoRon-mediated suppression of the aforementioned signalling pathways was abolished in podocytes with stable shRNA-mediated knockdown of AdipoR1 (ESM Fig. 4).

### AdipoRon attenuates LPS-induced migration and apoptosis of cultured podocytes

**Fig. 5:**
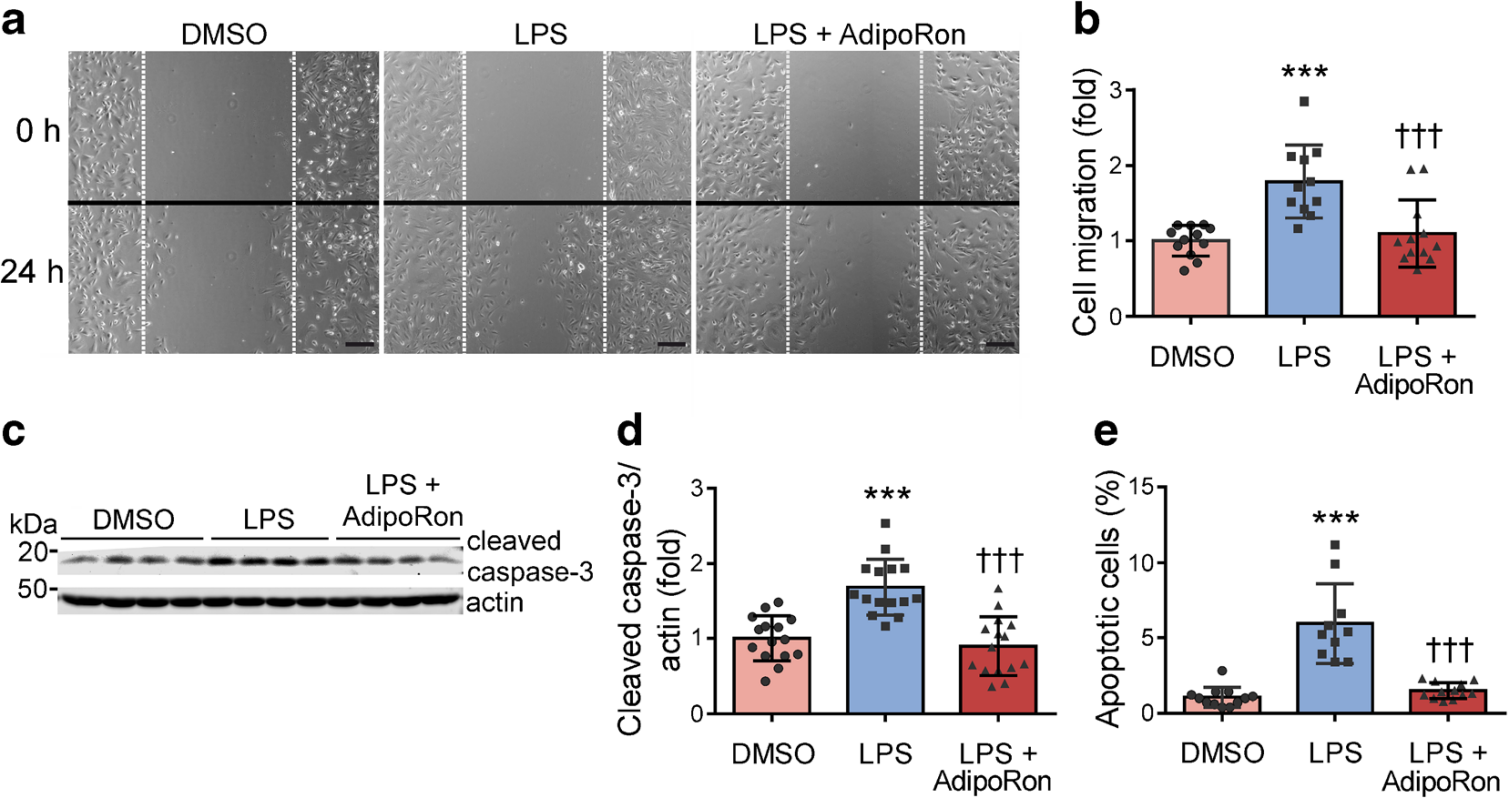
AdipoRon protects cultured human podocytes against LPS-induced migration and apoptosis. (**a**) Representative images of DMSO-, LPS- or LPS-AdipoRon-treated human podocytes taken at 0 h and 24 h after scratching the cell monolayer. Scale bar, 250 μm. (**b**) Quantification of the number of migrated cells into the scratched area after 24 h from three independent experiments. Each dot represents the data from one scratch. Results are normalised to DMSO-treated control. (**c**) Representative immunoblots of lysates prepared from human podocytes treated with AdipoRon or DMSO and stimulated with LPS for 48 h. (**d**) The expression level of cleaved caspase-3 normalised to actin quantified from four independent experiments as in (**c**). Results are normalised to DMSO-treated control. (**e**) The percentage of apoptotic annexinV-positive/7-AAD-negative cells measured by flow cytometry from three independent experiments. Data throughout were assessed by one-way ANOVA with a subsequent Bonferroni post hoc test. ***p < 0.001 vs DMSO. ^†††^*p* < 0.001 vs LPS

In vitro, stimulation with LPS increases migration of podocytes [24]. Using a scratch assay, we observed that AdipoRon attenuated LPS-induced podocyte migration by 39% (*p* < 0.001) (Fig. 5a,b). To investigate whether AdipoRon protects against LPS-induced podocyte apoptosis [20], we measured the expression of cleaved caspase-3 and the percentage of apoptotic annexinV-positive/7-AAD-negative cells. AdipoRon prevented LPS-induced apoptosis, as demonstrated by the reduction of the cleavage of caspase-3 by 47% (*p* < 0.001) (Fig. 5c,d) and the percentage of apoptotic cells by 75% (*p* < 0.001) (Fig. 5e).

### AdipoRon reduces LPS-induced secretion of proinflammatory cytokines in human glomeruli

**Fig. 6:**
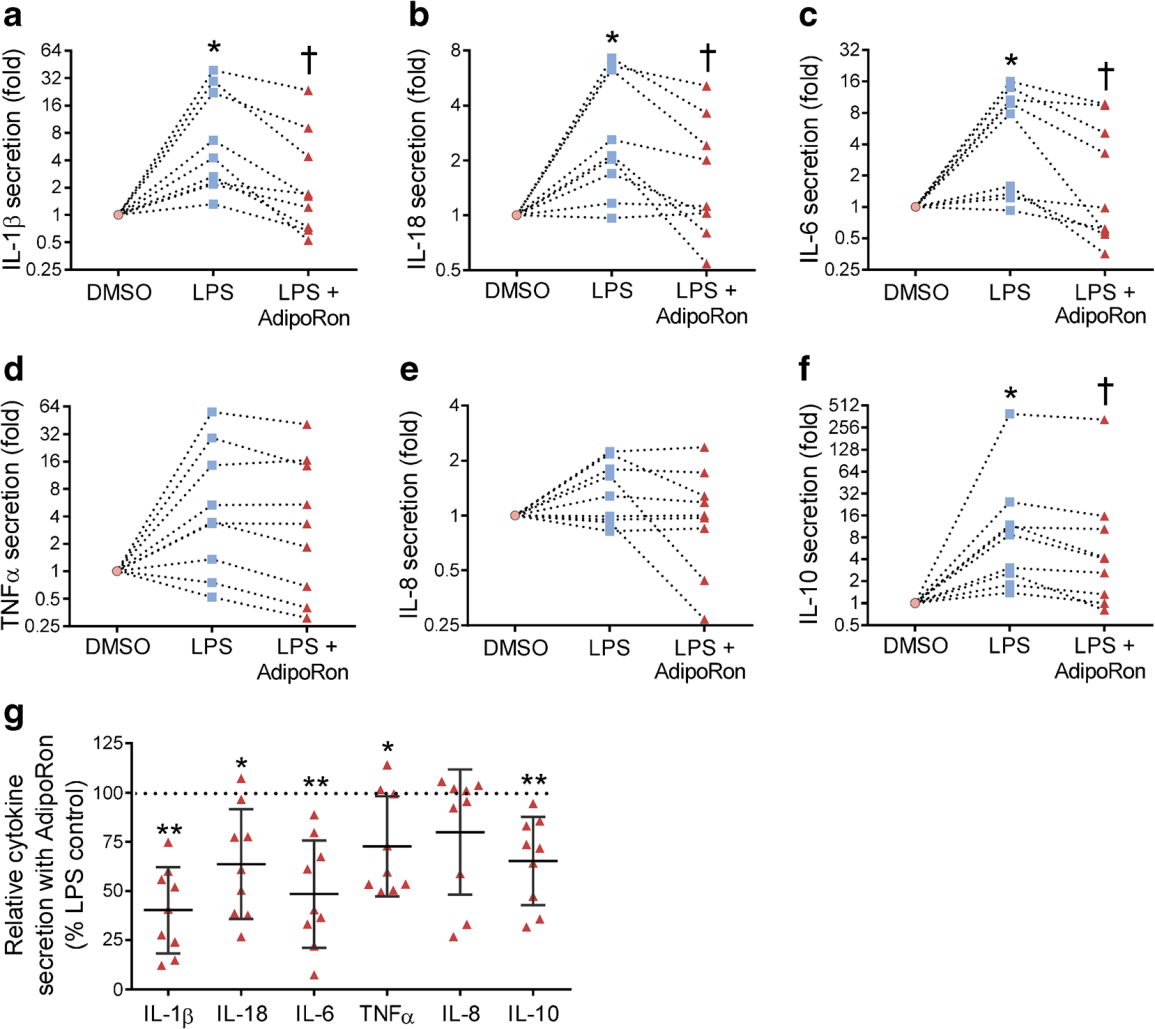
AdipoRon reduces the LPS-induced secretion of inflammatory cytokines in human glomeruli ex vivo*.* (**a**–**f**) Line plot graphs showing the fold change of IL-1β (**a**), IL-18 (**b**), IL-6 (**c**), TNFα (**d**), IL-8 (**e**) and IL-10 (**f**) secreted into the culture media of DMSO-, LPS- or LPS-AdipoRon-treated (24 h) glomeruli isolated from nine study participants. The *y*-axes are drawn using a log_2_ scale. The statistical differences between the treatment groups were analysed based on absolute cytokine concentrations (pg/ml) in culture media normalised to total glomerular protein (μg) using Friedman test for correlated samples with Bonferroni-adjusted post hoc Wilcoxon signed-rank test. *p < 0.05 vs DMSO. ^†^*p* < 0.05 vs LPS. (**g**) The relative cytokine secretion upon LPS-AdipoRon treatment expressed as % of LPS-only-treated glomeruli (set to 100%, horizontal dashed line). Data were assessed using one-sample Wilcoxon signed-rank test (against 100%). *p < 0.05 and **p < 0.01 vs LPS

To explore whether AdipoRon lowers LPS-induced inflammation in human glomeruli ex vivo, glomeruli isolated from renal cortex obtained from nephrectomies of nine study participants (ESM Table 2) were treated with DMSO, LPS or LPS-AdipoRon for 24 h. ESM Fig. 5 shows the cytokine secretion separately for each participant. The secretion of IL-1β, IL-18, IL-6 and IL-10 was increased by LPS and reduced by AdipoRon (Fig. 6a–c,f). The secretion of TNFα and IL-8 was upregulated by LPS in glomeruli of several participants (ESM Fig. 5d,e), but did not reach statistical significance in the collective analysis (Fig. 6d,e). In the analysis of the relative cytokine secretion upon LPS-AdipoRon treatment (% of LPS-treated), the secretion of IL-1β, IL-18, IL-6, TNFα and IL-10 was reduced (60 ± 22%, 37 ± 28%, 51 ± 27%, 27 ± 26% and 35 ± 22%, respectively, *p* < 0.05) (Fig. 6g). The LPS-induced cytokine secretion or the relative cytokine secretion with LPS-AdipoRon treatment (% of LPS-treated) did not differ between the participants with and without diabetes, or with BMI below or above 30 (data not shown).

Both the LPS-induced cytokine secretion (ESM Table 4) and the relative cytokine secretion upon LPS-AdipoRon treatment (% of LPS-treated) (ESM Table 5) showed a strong positive correlation between the studied cytokines. To evaluate whether the basal inflammatory status of the kidneys contributes to the cytokine response, we immunostained renal sections for TLR4, phospho-NF-κB-p65 and macrophage marker CD68 (ESM Fig. 6). The LPS-induced cytokine secretion did not correlate with the glomerular expression of TLR4 or phospho-NF-κB-p65, the glomerular number of CD68-positive macrophages, or BMI (ESM Table 4). Interestingly, the relative effect of AdipoRon on LPS-induced IL-6 secretion negatively correlated with the glomerular expression of TLR4, indicating that higher TLR4 expression resulted in reduced effect of AdipoRon on IL-6 secretion (ESM Table 5).

## Discussion

We show that AdipoRon ameliorates renal inflammation and injury in HFD-fed mice, attenuates LPS-induced inflammation and dysfunction in cultured human podocytes*,* and suppresses LPS-induced inflammatory cytokine secretion in isolated human glomeruli. Chronic low-grade inflammation precedes and promotes the progression of renal injury in obesity and diabetes [3–5, 25, 26]. Both conditions associate with increased serum activity of bacterial LPS [2, 27], which promotes chronic low-grade inflammation [2]. Taken together, our data indicate that activation of the adiponectin receptor may be a potent therapeutic strategy to ameliorate inflammation-related glomerular injury.

One of our main findings was that AdipoRon attenuated the glomerular expression of activated NF-κβ-p65, TNFα and IL-1β and the renal expression of IL-6 in HFD-fed obese mice. Studies on obese or diabetic animal models [5, 28–30] and clinical trials targeting proinflammatory molecules in diabetic kidney disease (DKD) [5] suggest that proinflammatory cytokines directly contribute to the progression of renal injury. Proinflammatory cytokines and related molecules are upregulated in the glomeruli of individuals with obesity- or diabetes-related kidney disease [31–33] and associate with the severity of diabetic glomerular injury [34, 35]. Interestingly, studies on obese individuals with pre-existing renal impairment undergoing bariatric surgery indicate that a reduction of renal inflammation may associate with improvements in renal function [3]. Renal upregulation of proinflammatory cytokines induces infiltration of macrophages, which further facilitates the inflammatory reaction. Accordingly, we observed increased glomerular infiltration of F4/80-positive macrophages in HFD-fed mice, and this was reduced by AdipoRon. Renal inflammation precedes and contributes to the development of glomerular fibrosis, which is an early sign of renal injury in obesity and diabetes and predicts the disease progression [1, 36]. Indeed, along with the reduction of glomerular fibronectin in HFD-fed mice, AdipoRon attenuated the glomerular expression of TGFβ and renal expression of IL-4, both known to promote renal fibrosis [6, 36, 37].

We also observed that AdipoRon ameliorates the structural changes of the glomerular filtration barrier, including glomerular hypertrophy, thickening of the GBM and podocyte foot process effacement (FPE). This is in line with previous studies on adiponectin-treated adiponectin-knockout mice [8] and AdipoRon-treated *db/db* mice [16]. Glomerular hypertrophy and thickening of the GBM are early renal manifestations of obesity- and diabetes-related kidney disease and precede albuminuria [1, 5]. FPE appears to precede podocyte detachment, and unlike podocyte detachment, it is a reversible process, which enables recovery of the normal podocyte structure if the local stress signals disappear [38]. In line with the reduced FPE, we observed that AdipoRon attenuated the LPS-induced migration of cultured podocytes, which is considered an in vitro analogue of FPE in vivo [38]. AdipoRon also prevented HFD-induced loss of glomerular podocytes, consistent with the protection against LPS-induced apoptosis of podocytes in vitro. Collectively, our study showed that AdipoRon reduces glomerular inflammatory signalling, expression of inflammatory cytokines and macrophage infiltration in HFD-fed mice, potentially contributing to the amelioration of glomerular injury.

AdipoRon also reduced HFD-induced weight gain in mice, which is in line with a previous study on corticosterone-induced obesity in mice [39] but in contrast to previous studies on *db/db* mice that exhibit, however, more severe metabolic dysfunction than HFD-fed mice [15, 16]. On the basis of the previous literature on adiponectin [40], we hypothesised that AdipoRon may prevent HFD-induced intestinal leakage, which potentially predisposes to renal injury [22]. Although the intestinal permeability was not upregulated by HFD in our DBA/2J mice as previously described in C57Bl6/J mice [21], HFD-fed mice showed an elevated serum LPS concentration. The unaltered intestinal permeability may be explained by the HFD-induced upregulation of intraluminal IAP activity, which detoxifies LPS and counteracts increased intraluminal LPS activity, potentially preventing intestinal leakage [21]. Genetic differences between the mouse strains may affect the sensitivity to developing intestinal dysfunction by regulating the capacity to maintain sufficient IAP activity when the intraluminal level of endotoxins is elevated [41, 42]. This may explain why HFD-induced intestinal leakage was not observed in the DBA/2J mice.

Stimulation of cultured podocytes with LPS revealed that the suppression of the central inflammatory signalling pathways, including NF-κB-p65, JNK and p38-MAPK, contributes to the anti-inflammatory effects of AdipoRon in an AdipoR1-dependent manner. As the renal expression of neither AdipoR1 nor TLR4 was altered by AdipoRon, our data suggest that the anti-inflammatory effects of AdipoRon are mediated, at least partly, by the activation of AdipoR1 and the downstream signalling pathways. Upregulation of the aforementioned signalling pathways is also observed in the glomeruli of individuals with obesity- and diabetes-related kidney disease [31, 32, 43–45]. These pathways contribute to renal fibrosis [4, 5], upregulation of proinflammatory cytokines and macrophage infiltration [4, 5], podocyte apoptosis [4, 6, 43] and derangement of the podocyte actin cytoskeleton, which is linked to increased podocyte migration [6]. In line with the suppressed inflammatory signalling, AdipoRon reduced the LPS-stimulated secretion of TNFα, migration and apoptosis in cultured podocytes. A reduced podocyte number is a strong predictor of albuminuria in obesity and diabetes [1, 6, 36], and apoptosis at least partly contributes to podocyte loss in DKD [43]. Taken together, our data suggest that the activation of AdipoR1 by AdipoRon attenuates podocyte injury via inhibition of the NF-κB-p65, JNK and p38-MAPK pathways and TNFα secretion.

A key finding of our study is that AdipoRon reduced the LPS-induced secretion of inflammatory cytokines in human glomeruli ex vivo, further highlighting the relevance of our data for inflammation-related renal injury in humans. Along with the downregulation of proinflammatory cytokines IL-1β, IL-18 and IL-6, AdipoRon downregulated the LPS-induced secretion of anti-inflammatory IL-10, which is secreted in response to proinflammatory cytokines in order to attenuate the established inflammatory reaction [46]. Indeed, reduction of IL-10 secretion by AdipoRon suggests that AdipoRon comprehensively reduces the glomerular inflammatory response. Although metabolic endotoxaemia contributes to chronic inflammation in obesity and diabetes, LPS-independent factors are also involved. Notably, the anti-inflammatory effects of AdipoRon are not limited to LPS-driven inflammation. AdipoRon suppresses proinflammatory cytokines in vitro in response to other inflammatory triggers as well [47–49], and attenuates inflammation in vivo in models of non-metabolic diseases [47, 48].

Our study has its limitations. The renoprotective effects of AdipoRon, including reduced glomerular expression of TGFβ and infiltration of macrophages, have been described in *db/db* mice, a model of advanced type 2 diabetes [16]. In that study, AdipoRon was shown to mediate its renoprotective effects through the Ca^2+^/liver kinase B1 (LKB1)–AMP-activated protein kinase (AMPK) /peroxisome proliferator-activated receptor α (PPAR-α) pathway, leading to a reduction in lipotoxicity and oxidative stress [16]. Our study expands the previous data in multiple ways. First, we analysed the renal expression of numerous central proinflammatory molecules in HFD-fed mice. Second, we identified the intracellular signalling pathways that mediate the anti-inflammatory effects of AdipoRon in glomerular podocytes in vitro. Third, by demonstrating that AdipoRon reduced the secretion of inflammatory cytokines in LPS-stimulated human glomeruli, we confirmed that the anti-inflammatory effects of AdipoRon are mediated via lowering local glomerular inflammation, thus excluding the effects of systemic inflammation. Importantly, isolated human glomeruli provide several advantages over cell lines and animal models, as they retain their original donor-related morphological and functional characteristics, and therefore, better reflect the in vivo environment of the human kidney.

Collectively, our data indicate that the renoprotective effects of AdipoRon in mice receiving HFD are mediated via lowering inflammatory signalling and the secretion of proinflammatory cytokines in the glomerular cells. Inflammation is a potential therapeutic target in both obesity- and diabetes-related kidney disease. Due to the pleiotropic nature of cytokines, targeted inhibition of individual cytokines with, for example, monoclonal antibodies may disturb their complex interplay and thereby cause adverse effects, such as an impaired host defence. Hence, a better strategy could be to target the molecules that exert wide anti-inflammatory actions through effects on several signalling pathways and cytokines, such as the adiponectin signalling pathway. Furthermore, the manufacturing of biologics is expensive compared with chemical drugs. AdipoRon has emerged as a promising drug candidate for a wide range of diseases [50], and our study proposes that activating the adiponectin receptor by AdipoRon is a potent strategy to alleviate inflammation-related renal injury.

## Supplementary Information

ESM (PDF 1.75 MB)

## Data Availability

All data generated and analysed during this study are available from the corresponding author on reasonable request.

## Abbreviations

7-AAD: 7-Aminoactinomycin D
AdipoR1/2: Adiponectin receptor 1/2
DKD: Diabetic kidney disease
FPE: Foot process effacement
GBM: Glomerular basement membrane
HFD: High-fat diet
IAP: Intestinal alkaline phosphatase
IκBα: NF-kappa-B inhibitor alpha
JNK: c-Jun N-terminal kinase
LFD: Low-fat diet
LPS: Lipopolysaccharide
OCT: Optimal cutting temperature
p38-MAPK: p38 Mitogen-activated protein kinase
PAS: Periodic acid–Schiff
shRNA: Short hairpin RNA
TLR4: Toll-like receptor 4
WT1: Wilms’ tumour protein
