# Supplementary material for: Adiponectin receptor agonist AdipoRon ameliorates renal inflammation in diet-induced obese mice and endotoxin-treated human glomeruli ex vivo
Source: Diabetologia. 2021 May 14;64(8):1866–79. doi: 10.1007/s00125-021-05473-9 (PMC8245393; doi:10.1007/s00125-021-05473-9)
Supplement: Supplementary file 1 — (PDF 1.75 MB) [file 125_2021_5473_MOESM1_ESM.pdf]

Lindfors et al., Electronic Supporting Material (ESM)

ESM Methods

Reagents RPMI-1640 (#R0883), FBS (#F7524), penicillin/streptomycin, *Escherichia coli* 0111:B4 LPS (#L2630), AdipoRon (#SML0998, cell studies) and DMSO were from MilliporeSigma; AdipoRon (#Z32352521, mouse study) from Enamine (Kiev, Ukraine); ultraglutamine from Lonza (Verviers, Belgium); DMEM (#BE12-707F) from Lonza (Walkersville, MD, USA); Insulin-Transferrin-Selenium (ITS) from Gibco (St. Louis, MO, USA) and Complete Protease Inhibitor Cocktail from Roche Diagnostics (Mannheim, Germany). Antibodies (ESM Table 1) were validated by knockout/overexpression experiments or passed the manufacturer's application-specific testing standards.

Animals Five-week-old male DBA/2J mice were purchased from Charles River Laboratories (Lyon, France). Mice were maintained on a 12 h light-dark cycle and humidity of 55±15% at 22±2ºC in individually ventilated cages in the conventional unit at the Laboratory Animal Centre of the University of Helsinki. Cages were enriched with aspen bedding, a wooden stick, nesting material and a nest box. Experimental procedures were performed during the light cycle. The experiments were performed according to the Federation of European Laboratory Animal Science Associations (FELASA) guidelines (2015) and approved by the National Animal Experiment Board**.**

After acclimation for 1 week on a regular chow diet, mice were randomly assigned to experimental groups (2 mice/cage) based on body weight, and fed with either low-fat diet (LFD; 10% kJ fat, #D12450B, Research Diets, NJ, USA) (n=8), high-fat diet (HFD; 60% kJ fat, #D12492, Research Diets) (n=14), or HFD supplemented with AdipoRon (380 mg/kg diet, custom-made by Research Diets) (n=14), for 9 weeks, with ad libitum access to water and diet. The dose was chosen based on previous studies administering AdipoRon 30–50 mg/kg body weight [1, 2], yielding a mean dose of AdipoRon 51.5 mg/kg at the beginning and 28.2 mg/kg at the end of the experiment, as the mice gained weight. A pilot study was performed to determine the statistically relevant number of animals.

Body weight and food intake (per two mice) were monitored weekly. After 7 weeks of treatment, blood glucose was measured from the tail vein blood with Glucometer Elite (Bayer, Leverkusen, Germany) after 6 h fasting. At the baseline and after 8 weeks of treatment, mice were kept individually in metabolic cages for 24 h with free access to food and water, and urine and faecal pellets were collected.

Harvesting of organs After 9 weeks of treatment, mice were killed by CO_2 inhalation followed by cervical dislocation, and tissues were collected. Serum samples were prepared from blood obtained by cardiac puncture. Renal cortex and intestinal tissue were snap-frozen with liquid nitrogen and stored at -80°C. Part of renal cortex was also fixed overnight in 10% (vol./vol.) formalin followed by paraffin embedding or embedded in Tissue-Tek optimal cutting temperature compound (OCT; Sakura Finetek, Torrance, CA, USA) followed by snap-freezing on the surface of liquid nitrogen. Renal tissue was homogenised in a glass homogeniser with 1% (vol./vol.) Igepal, 150 mmol/l NaCl, 20 mmol/l TRIS-HCl, pH 7.5 supplemented with 1x proteinase-inhibitor cocktail, 50 mmol/l sodium fluoride and 1 mmol/l sodium orthovanadate. Protein concentration was measured with Bradford Protein Assay Dye Reagent (Bio-Rad, Hercules, CA, USA). For transmission electron microscopy, several 1 mm × 1 mm × 1 mm cubes from renal cortex were cut and processed as described [3]. Briefly, tissue was fixed in 1.5% (vol./vol.) glutaraldehyde, 3% (wt/vol.) paraformaldehyde, 5% (wt/vol.) sucrose in 100 mmol/l phosphate buffer pH 7.4 for 2 h, postfixed in 1% OsO4 (wt/vol.) in the same buffer 1 h, stained *en-block* in 1% (wt/vol.) uranyl acetate in 10% ethanol for 1 h, dehydrated in ethanol and embedded in LX-112 resin.

Serum and urine biochemical analysis Serum adiponectin concentration was measured by mouse Adiponectin/Acrp30 Quantikine ELISA kit (#MRP300, R&D Systems, Minneapolis, MN, USA) according to the instructions from the manufacturer. Serum insulin concentration was measured by Ultra Sensitive Mouse Insulin ELISA kit (#90080, Crystal Chem, Chicago, IL, USA) according to the instructions from the manufacturer. Serum LPS concentration was measured by ELISA kit (#CSB-E13066m, Cusabio Biotech, Wuhan, China) according to the instructions from the manufacturer.

Immunoblotting Expression of AdipoR1, toll-like receptor 4 (TLR4), cleaved caspase-3 and the phosphorylated forms of NF-kappa-B inhibitor alpha (IκBα), NF-κB-p65, c-Jun N-terminal kinase (JNK) and p38 mitogen activated protein kinase (p38-MAPK) was studied by immunoblotting. Equal amounts of protein samples prepared from tissue (75 µg) or cellular extracts (20-50 µg) were separated by SDS-PAGE and transferred to PVDF-FL membranes (Millipore, Billerica, MA, USA). Membranes were blocked with Odyssey blocking buffer (TBS) (LI-COR, Lincoln, NE, USA) diluted 1:1 with TRIS-buffered saline and incubated with primary antibodies (in blocking buffer supplemented with 0.1% [vol./vol.] Tween-20) and secondary antibodies (in blocking buffer supplemented with 0.1% [vol./vol.] Tween-20 and 0.01% [wt/vol.] SDS). For antibody details, see ESM Table 1. Membranes were imaged with Odyssey Infrared Imaging System (LI-COR) and data

analysed using Image Studio software (LI-COR). Equivalent protein loading was ensured by normalising the target protein signal to actin or tubulin.

Iohexol experiment for intestinal permeability After 6 weeks of treatment, eight mice randomly selected from each group were tested for intestinal permeability by the iohexol assay as described [4]. Briefly, mice were administered 4 ml/kg iohexol (Omnipaque 300, GE Healthcare, Oslo, Norway) by oral gavage and placed into metabolic cages for urine collection (24 h). Urinary iohexol concentration was measured by ELISA kit (BioPAL, Worcester, MA, USA) according to the instructions from the manufacturer. The results are presented as percentage of excreted iohexol in the urine during 24 h (mg) per amount of administered iohexol (mg) as described [4]. After the urine collection, the number of faecal pellets adhered to the bottom grid of the metabolic cages were counted, and this was presented as the diarrhoea index (n=4 for each group).

Faecal albumin assay Faecal pellets collected in metabolic cages (24 h) after 8 weeks of treatment were extracted in 100 mmol/l PBS pH 7.4, 0.1% (vol./vol.) Tween-20 (100 mg of faecal/ml of buffer) supplemented with 1x protease inhibitor cocktail (Roche Diagnostics). Faecal albumin concentration was measured from soluble extractions obtained after centrifugation at 10,000 g for 10 min by mouse albumin ELISA kit (#KSP-170, Nordic BioSite, Täby, Sweden) according to the instructions from the manufacturer.

Intestinal alkaline phosphatase assay The intraluminal contents of caecum and distal colon were homogenised in extraction buffer (0.1 mg of intestinal content/µl of buffer: 0.1 mmol/l $ZnCl_2$, 1 mmol/l $MgCl_2$, 10 mmol/l TRIS-HCl pH 8.0) containing 1x EDTA-free protease inhibitor cocktail (Roche Diagnostics) with 0.1 mm glass beads (Precellys, Bergin Technologies, Montigny, France) followed by centrifugation at 11,150 g for 10 min at +4°C. Supernatants were subjected to determinations of total protein concentration by the Lowry method (DC protein assay, Bio-Rad). Intestinal alkaline phosphatase (IAP) activity in the supernatants was determined by an in-house colorimetric p-nitrophenyl phosphate (pNPP) assay exactly as described [5]. Briefly, standard curve was created by preparing serial dilutions of pNPP (MilliporeSigma) and 1:500 diluted CIAP (10,000 U/ml). Sample reactions (100 µl) constituted of 10 µl diluted faecal supernatant, 45 µl assay buffer (0.1 mmol/l ZnCl2, 1 mmol/l MgCl2, 10 mmol/l TRIS-HCl pH 10.0) and 45 µl 4.56 mmol/l pNPP stock. Standards and samples were incubated at +37°C for 30 min. The reaction was stopped by adding 20 µl 3 mol/l NaOH and absorbances 405/630 nm were measured. The IAP activities were calculated with the following formula: IAP activity (U/ml) = A/V/T where A is the amount of pNP

generated in μmol, V is the sample volume in ml and T is the reaction time in minutes. The final IAP activities were normalised to the faecal protein concentrations.

Immunohistochemistry and morphometric analysis After fixation, tissue was embedded in paraffin at the Tissue Preparation and Histochemistry Unit Meilahti (mouse tissue; University of Helsinki) or at the Laboratory of Pathology (human tissue; HUSLAB). Four-micrometre-thick sections were prepared for immunohistochemistry or periodic acid–Schiff (PAS) staining using standard procedures. Antigen retrieval for deparaffinised renal sections was carried out using 10 mmol/l citrate buffer (pH 6.0), 10 mmol/l TRIS-EDTA (pH 9.0) or EnVision FLEX Target Retrieval Solution (Dako, Santa Clara, USA) as indicated in ESM Table 1 in either PT module (Thermo Scientific, Runcorn, UK) or Decloaking Chamber NxGen (Biocare Medical, Pacheco, CA, USA). Sections were incubated with primary antibodies against TGFβ, TNFα, IL-1β, phospho-NF-κB-p65, F4/80, fibronectin and Wilms' tumour protein (WT1) diluted in Dako REAL Antibody Diluent (#S2022, Dako) as indicated in ESM Table 1. Normal rabbit IgG (#02-6102, Invitrogen) or mouse IgG (#026502, Invitrogen) were used as negative controls. Detection was performed using Brightvision Poly-HRP-anti-Rabbit IgG kit (mouse tissue; ImmunoLogic, Amsterdam, the Netherlands), EnVision+ immunohistochemistry kit (mouse tissue; Dako) or EnVision FLEX+ immunohistochemistry kit (human tissue; Dako) and 3,3'-diaminobenzidine (Dako) as indicated in ESM Table 1. Slides were counterstained with Mayer's Hematoxylin (Dako). Images were obtained using 3DHISTECH Pannoramic 250 FLASH II digital slide scanner (Genome Biology Unit supported by HiLIFE and the Faculty of Medicine, University of Helsinki, and Biocenter Finland) at 20× magnification. Glomerular staining area and total glomerular tuft area in PAS-stained sections was quantified from 15-20 glomeruli/section using the Histoquant module of the QuantCenter software version 2.0 (3DHISTECH, Budapest, Hungary). For podocyte count, WT1-positive nuclei were counted from glomeruli larger than 70 μm in diameter to ensure sectioning through the center of the glomerulus.

Cytokine measurements Cytokines were measured from renal cortex lysates (IL-4, IL-6) and culture media supernatants of podocytes or isolated human glomeruli (IL-1β, IL-6, IL-8, IL-10, IL-18, TNFα) with multiplex Q-Plex cytokine assays (Quansys Biosciences, Logan, UT, USA). Renal tissue was homogenised in a glass homogeniser with 1% (vol./vol.) Igepal, 150 mmol/l NaCl, 20 mmol/l TRIS-HCl pH 7.5 supplemented with 1x proteinase-inhibitor cocktail, 50 mmol/l sodium fluoride and 1 mmol/l sodium orthovanadate. Collected culture media samples were centrifuged at 1000 g for 5 min at +4°C in order to get rid of any floating cells. Cytokine assays were performed according to the

instructions from the manufacturer. Renal cortex samples were diluted 1:2. Culture media supernatants were assayed neat or diluted from 1:2 to 1:100 depending on the sample and cytokine assayed. Luminometric signal was measured by Q-View Imager LS camera (Quansys Biosciences) and data analysed by Q-View software (Quansys Biosciences).

Macrophage polarisation analysis Double immunofluorescence staining was performed on frozen OCT-embedded renal cortical sections against CD16/32 (M1 marker) or CD206 (M2 marker) and F4/80 (total macrophage marker). Sections (5-µm) were fixed in 4% (wt/vol.) paraformaldehyde, permeabilised with 0.1% (vol./vol.) Triton X-100 in PBS, blocked with CAS block, incubated with primary antibodies (F4/80 and CD16/CD32 or CD206) diluted in Dako REAL Antibody Diluent (Dako) for overnight at +4°C, incubated with secondary antibodies together with nucleic acid stain Hoechst 33342 for 1 h, and mounted in Prolong Diamond Antifade Mountant (Invitrogen). Sections incubated only with Dako REAL Antibody Diluent were used as negative controls. The images were generated using 3DHISTECH Pannoramic 250 FLASH II digital slide scanner (Genome Biology Unit supported by HiLIFE and the Faculty of Medicine, University of Helsinki, and Biocenter Finland) at 40× magnification using the filters for DAPI, FITC 38 HE and mPlum 64 HE.

The images were analysed with CellProfiler (version 3.1.8) [6] as follows: The illumination correction function was calculated and then applied to each image representing the entire stained tissue section using a background method and smoothing Gaussian Filter. To count the M2-polarized macrophages, the main steps of the pipeline included: 1) A manual global threshold applied respectively to the F4/80- and CD206-stained images; 2) Detection of the nuclei using Hoechst 33342-stained images; 3) Detection of the cell borders by expansion of the nuclei; 4) Identification of the macrophages by filtering out cells with a F4/80-integrated pixel intensity under 10; and 5) Detection of the M2-polarized macrophages by selecting macrophages with a CD206 integrated pixel intensity above 0.

To count the M1-polarized macrophages, the pipeline included the steps described above with additional steps to exclude unspecific staining: 1) A Gaussian filter was applied to the F4/80-stained images; 2) The image intensity was then measured and the median intensity was subtracted from the original F4/80-stained images; 3) A manual global threshold was applied respectively to the F4/80- and CD16/CD32-stained images; 4) The former was dilated using a disk shape element of 1 pixel; and 5) The dilated images were then used to mask the F4/80-negative regions in the CD16/32-stained images. Nuclei, cell borders and CD16/CD32 positive cells were detected as aforementioned.

Electron microscopy Transmission electron microscopy images were obtained and analysed as described [3] (*n*=3 mice for each group). Briefly, thin sections were stained with uranyl acetate and lead citrate, examined under 80 kV with JEM-1400 Transmission Electron Microscope (Jeol, Tokyo, Japan) and imaged at a magnification of 5000× and 30,000× with Olympus-SIS Morada digital camera (Olympus Soft Imaging Solutions GmbH, Münster, Germany). Images were analysed with Fiji ImageJ software version 1.52 (National Institutes of Health, Bethesda, MD, USA). Mean podocyte foot process width was analysed from 5–7 glomeruli/mouse (at least 18 image fields/mouse at 5000× magnification) and the thickness of the glomerular basement membrane (GBM) from 3 glomeruli/mouse (at least 10 image fields/mouse at 30 000× magnification).

Cell culture and treatments Conditionally immortalised human podocytes AB8/13 [7] (provided by M. Saleem [Southmead Hospital, University of Bristol, Bristol, UK]) were maintained at 33°C (5% CO_2) in RPMI-1640 (2 g/l glucose) supplemented with 10% (vol./vol.) FBS, 100 U/ml penicillin, 100 µg/ml streptomycin, 2 mmol/l glutamine and 1% (vol./vol.) ITS. Podocytes were differentiated at 37°C for 12–14 days, pre-treated with 12 µmol/l AdipoRon (maximum serum concentration in AdipoRon-administered mice [1]) or DMSO for 2 h, with subsequent stimulation with 200 ng/ml LPS for 1 h (phospho-proteins), 24 h (migration, cytokines) or 48 h (apoptosis). Cells were tested to be mycoplasma-negative using VenorGeM Classic Mycoplasma Detection Kit (Minerva Biolabs, Berlin, Germany). Cells were lysed in 1% (vol./vol.) Igepal, 0.5% (wt/vol.) sodium deoxycholate, 0.1% (wt/vol.) SDS, 150 mmol/l NaCl, 50 mmol/l TRIS-HCl pH 8.0 supplemented with 1x proteinase-inhibitor cocktail, 50 mmol/l sodium fluoride and 1 mmol/l sodium orthovanadate. Protein concentration was measured with Bradford Protein Assay Dye Reagent (Bio-Rad).

Generation of stable AdipoR1 knockdown podocyte line Stable AdipoR1 knockdown human podocyte cell line was generated by lentiviral delivery of human *ADIPOR1* shRNA (clone TRCN000063043) in a pLKO.1 vector obtained from TRC1 shRNA library (Biomedicum Functional Genomics Unit (FuGU), University of Helsinki) into proliferating AB8/13 podocytes. Lentivirus was produced by co-transfecting HEK293FT cells (Invitrogen) with lentiviral packaging constructs CMVDelta8.9 (FuGu) and phCMVg (FuGu), together with *ADIPOR1* shRNA or non-target shRNA (scramble) pLKO.1 vector, with Lipofectamine 2000 (Invitrogen). HEK293FT cells were maintained at 37°C (5% CO_2) in DMEM (#6546, MilliporeSigma) containing 4.5 g/l glucose supplemented with 10% (vol./vol.) FBS, 100 U/ml penicillin, 100 µg/ml streptomycin, 2 mmol/l glutamine, 1 mmol/l sodium pyruvate (Gibco) and 1x MEM Non-Essential Amino Acids Solution (#11-140-050, Gibco). After 72 h transfection, virus-containing media was filtrated through 0.45 µm filter and

ultracentrifuged at 85,000 g for 90 min at +4 °C. Proliferating podocytes were infected with lentivirus resuspended in PBS and selected with 1 µg/ml puromycin (InvivoGen, San Diego, CA) for 9 days. The selected puromycin concentration was determined by titrating the lowest concentration that killed 100% of the nontransduced control cells after 48 h. The efficiency of knockdown was verified by immunoblotting (ESM Fig. 4a–b).

Migration assay Podocyte migration was measured by in vitro scratch assay as described [8]. Before starting the treatments, a scratch was made on a confluent cell monolayer by using a 200-µl pipette tip, and the cells were shortly washed once with complete culture media. The plate was imaged at 0 h and after 24 h of treatment with Cytation5 Multi-Mode Reader (BioTek, Winooski, VT, USA) at 4× magnification. The number of migrated cells was counted from captured images (*n*=3–4 scratches per each treatment in each experiment).

Apoptosis assay Podocyte apoptosis was measured by double staining for annexinV-FITC and 7-aminoactinomycin D (7-AAD) (BD Biosciences, Franklin Lakes, NJ, USA) as described [9]. After indicated treatments, cells were washed with PBS, re-suspended in 1x AnnexinV Binding Buffer (100 µl per cells collected from two wells of a 6-well plate) (BD Biosciences) and incubated with annexinV-FITC (5 µl) (#556419, BD Biosciences) and 7-AAD (5 µl) (BD Biosciences) for 15 min at room temperature. After staining, 800 µl of 1x AnnexinV Binding Buffer was added to each sample, and the cells were filtered through a 100-µm cell strainer. A minimum of 1.5×10^4 cells was counted by Accuri C6 flow cytometer (BD Biosciences). Cells positive for annexinV-FITC but negative for 7-AAD were defined as apoptotic.

Statistics Statistical analyses were performed using SPSS version 25.0 (IBM, Chicago, IL, USA) and data presented as mean±SD unless otherwise specified. To compare means between two groups, unpaired two-tailed Student’s t test was performed. To compare means between three groups, one-way ANOVA (normal distribution) or Kruskal Wallis test (non-normal distribution) with Bonferroni (equal variances) or Tamhane (unequal variances) post hoc test was performed. Mouse weight gain was analysed by two-way repeated measures ANOVA using GraphPad Prism software version 9.0 (San Diego, CA, USA). The differences in cytokine secretion (absolute concentration [pg/ml]/total protein [µg]) between the treatment groups of human glomeruli were analysed using non-parametric Friedman test for three-group comparison of correlated samples with Bonferroni-adjusted post hoc Wilcoxon signed-rank test. The relative effect of LPS-AdipoRon treatment in comparison to corresponding LPS control (set to 100%) was evaluated using one-sample Wilcoxon signed-rank test

(against 100%). Bivariate correlations were analysed with Pearson's test. A p value < 0.05 was considered statistically significant.

In vitro experiments included 3–5 independent experiments with 3–4 biological replicates/treatment. Each dot represents the data obtained from one mouse or one well in a multi-well plate unless otherwise stated. Image analysis of the tissue sections was performed blindly by one investigator. For the iohexol assay, eight mice were randomly selected from each group. Otherwise randomization and blinding were not carried out.

Criteria for data exclusions were: detached tissue section (one HFD mouse in Fig. 3b), absence of renal cortex tissue in the tissue block (one HFD-AdipoRon mouse in ESM Fig. 3a, b), broken tissue slide (one HFD mouse in Fig. 3b; one HFD-AdipoRon mouse in Fig. 3i) or obvious lack of primary antibody (one HFD-AdipoRon mouse in Fig. 3c) during IHC staining, as well as too small amount of intestinal contents for extractions (one LFD mouse, seven HFD mice and eight HFD-AdipoRon mice in ESM Fig. 2d). One LFD mouse was prematurely killed for welfare issues and excluded from the study.

ESM Table 1. Antibodies used for immunoblotting (IB) and immunohistochemistry (IHC) with corresponding antigen retrieval buffers and detection kits used in IHC. CB, Citrate buffer; TE, TRIS-EDTA buffer; EF, EnVision FLEX Target Retrieval Solution; EnVision+, EnVision+ immunohistochemistry kit; FLEX+, EnVision FLEX+ immunohistochemistry kit

**Primary antibodies**	**Manufacturer**	**Catalog #**	**RRID**	**Host**	**Dilution IB**	**Dilution IHC**	**Retrieval buffer / Detection kit**
Fibronectin	Abcam, Cambridge, UK	299	303474	rabbit		1:500	CB / Brightvision
TGFβ1 [a]	Abcam	215715	N/A	rabbit		1:100	EF / EnVision+
F4/80	Cell Signaling Technology (CST), Beverly, MA, USA	70076	2799771	rabbit		1:100	EF / EnVision+
CD68	CST	76437	2799882	rabbit		1:400	CB / FLEX+
Phospho-JNK	CST	9251	331659	rabbit	1:1000		
Phospho-p38 MAPK	CST	4511	2139682	rabbit	1:1000		
NF-κB-p65	CST	8242	10859369	rabbit	1:1000		
Phospho-NF-κB-p65	CST	3037	2341216	rabbit	1:1000	1:100	TE / Brightvision (for mouse), FLEX+ (for human)
p38 MAPK	CST	9212	330713	rabbit	1:1000		
Cleaved caspase-3	CST	9661	2341188	rabbit	1:1000		
Phospho-NF-κB-p65	CST	3036	331281	mouse	1:1000		
Phospho-IκBα	CST	9246	2267145	mouse	1:1000		
Actin	MilliporeSigma, Burlington, MA, USA	A4700	476730	mouse	1:2000		
Tubulin	MilliporeSigma	T6199	477583	mouse	1:3000		
JNK	R&D Systems, Minneapolis, MN, USA	MAB1387	2297386	mouse	1:1000		
TLR4	Santa Cruz Biotechnology, Santa Cruz, CA, USA	293072	10611320	mouse		1:500	CB / FLEX+
TNFα	Novus Biologicals, Littleton, CO, USA	NBP1-19532	1643202	rabbit		1:300	CB / Brightvision

IL-1β	Rockland Immunochemicals, Gilbertsville, PA, USA	210-401-319	10704513	rabbit		1:120	CB / Brightvision
WT1	Santa Cruz Biotechnology	192	632611	rabbit		1:1000	CB / Brightvision
AdipoR1 [b]	Immuno-Biological Laboratories (IBL), Takasaki, Japan	18993	N/A		1:200		
CD206 (MMR)	R&D Systems, Minneapolis, MN, USA	AF2535	2063012	goat		1:50	
CD16/CD32	BD Biosciences, San Diego, CA, USA	553141	394656	rat		1:7	
**Secondary antibodies**							
Alexa Fluor 680 anti-rabbit IgG	Invitrogen, Carlsbad, CE, USA	A10043	2534018	donkey	1:10,000		
IRDye 680 anti-Rabbit IgG	LI-COR, Lincoln, NE, USA	926-32221	621841	goat	1:10,000		
IRDye 680RD anti-mouse IgG	LI-COR	926-68072	10953628	donkey	1:10,000		
IRDye 800CW anti-rabbit IgG	LI-COR	926-32213	621848	donkey	1:10,000		
IRDye 800CW anti-mouse IgG	LI-COR	926-32212	621847	donkey	1:10,000		
Alexa Fluor 488 anti-rat IgG	Invitrogen	A-21208	2535794	donkey		1:1000	
Alexa Fluor 594 anti-rabbit IgG	Invitrogen	A-21207	141637	donkey		1:1000	
Alexa Fluor 488 anti-goat IgG	Invitrogen	A-11055	2534102	donkey		1:1000	

[a] Knockout-validated by the manufacturer

[b] Knockdown-validated in ESM Fig. 4a,b and knockout/overexpression-validated by Sluch et al. [10]

ESM Table 2. Characteristics of the study participants used for the isolation of glomeruli and analysis of LPS-induced cytokine secretion. T2D, type 2 diabetes

ID	T2D	BMI	Sex	Age, years	Other relevant medical conditions	Medication
**#1**	No	20.1	F	68	Hypertension, hypercholesterolemia	Amlodipine, bisoprolol, hydrochlorothiazide, simvastatin
**#2**	No	22.2	M	81	Hypertension	Candesartan, furosemide, simvastatin, tinzaparin sodium, warfarin
**#3**	No	25.5	M	78	Hypertension, hypercholesterolemia	Amlodipine, hydrochlorothiazide, losartan, metoprolol, simvastatin, tinzaparin sodium
**#4**	No	30.1	F	72	Hypertension	Telmisartan
**#5**	No	42.5	F	64	Hypertension, hypercholesterolemia hyperthyreosis	Atorvastatin, levothyroxine, prednisolone
**#6**	Yes	19.6	F	83	Hypertension, hypothyreosis, proteinuria (U-prot 176 mg/l), normal eGFR (62 ml/min/1.73 m^2)	Amlodipine, enalapril, hydrochlorothiazide, levothyroxine, metformin, metoprolol, simvastatin
**#7**	Yes	24.4	M	61	Hypertension, diabetic neuropathy	Amlodipine, bisoprolol, enoxaparin sodium, etoricoxib, hydrochlorothiazide, insulin, lisinopril, simvastatin
**#8**	Yes	31.2	F	63	Hypertension, hypercholesterolemia	Atorvastatin, enalapril, metformin
**#9**	Yes	42.6	M	77	Hypertension, no proteinuria, reduced eGFR (50 ml/min/1.73 m^2)	Apixaban, atorvastatin, bisoprolol, furosemide, insulin, prednisolone, perindopril

ESM Table 3. General parameters of the mice. Data is shown as mean ± SD. Statistical analysis was performed using one-way ANOVA with a subsequent Bonferroni post hoc test or by Kruskal Wallis test, due to non-normal distribution, with a subsequent Bonferroni post hoc test. LFD, n=7; HFD, n=14; HFD-AdipoRon, n=14. *p < 0.05, **p < 0.01 and ***p < 0.001 versus LFD. LFD, low fat diet; HFD, high fat diet

	**LFD**<br>**Mean ± SD**	**HFD**<br>**Mean ± SD**	**HFD + AdipoRon**<br>**Mean ± SD**
24 h urine albumin excretion (µg)	6.60 ± 3.24	18.52 ± 12.14 *	28.93 ± 33.72 *
24 h urine albumin/creatinine (mg/mmol)	6.49 ± 5.01	8.52 ± 6.08	12.59 ± 11.98
24 h urine volume (µl)	255.71 ± 169.10	399.29 ± 171.43	274.64 ± 171.11
Kidney weight (g)	0.25 ± 0.02	0.29 ± 0.02 ***	0.28 ± 0.03
Serum adiponectin (ng/ml)	6.27 ± 1.17	5.06 ± 0.66 **	4.79 ± 0.58 ***
Non-fasting serum insulin (pmol/l)	364.15 ± 176.67	1022.00 ± 779.37 *	856.63 ± 764.42
Fasting blood glucose (mmol/l)	6.96 ± 0.32	8.29 ± 0.90 ***	7.79 ± 0.90

ESM Table 4. Bivariate Pearson’s correlation coefficients (*r*) for LPS-induced cytokine secretion in human glomeruli ex vivo (expressed as fold change to DMSO control), BMI values of the study participants, and baseline expression levels of inflammation markers in glomeruli studied by immunohistochemistry (IHC). **$*p < 0.05$** and **$**p < 0.01$**

		BMI	TLR4 (IHC)	p-NFκB (IHC)	CD68 (IHC)	IL-1β (LPS)	IL-6 (LPS)	IL-8 (LPS)	IL-10 (LPS)	IL-18 (LPS)	TNFα (LPS)
**BMI**	*r*	1									
	p										
**TLR4 (IHC)**	*r*	-0.380	1								
	p	0.314									
**p-NFκB (IHC)**	*r*	-0.225	0.690*	1							
	p	0.560	**0.040**								
**CD68 (IHC)**	*r*	-0.457	0.389	0.627	1						
	p	0.216	0.300	0.071							
**IL-1β (LPS)**	*r*	0.365	-0.633	-0.220	-0.195	1					
	p	0.333	0.067	0.569	0.615						
**IL-6 (LPS)**	*r*	0.488	-0.272	-0.043	-0.392	0.732*	1				
	p	0.183	0.479	0.913	0.297	**0.025**					
**IL-8 (LPS)**	*r*	0.451	-0.398	-0.139	-0.241	0.911**	0.871**	1			
	p	0.224	0.288	0.721	0.531	**0.001**	**0.002**				
**IL-10 (LPS)**	*r*	0.103	-0.014	0.177	-0.219	0.276	0.594	0.282	1		
	p	0.792	0.972	0.648	0.571	0.472	0.092	0.463			
**IL-18 (LPS)**	*r*	0.164	-0.524	-0.117	-0.110	0.913**	0.766*	0.814**	0.576	1	
	p	0.674	0.147	0.763	0.777	**0.001**	**0.016**	**0.008**	0.105		
**TNFα (LPS)**	*r*	0.100	-0.233	0.053	-0.258	0.623	0.828**	0.625	0.876**	0.846**	1
	p	0.799	0.547	0.892	0.503	0.073	**0.006**	0.072	**0.002**	**0.004**	

ESM Table 5. Bivariate Pearson's correlation coefficients (*r*) for relative cytokine secretion with LPS-AdipoRon treatment (% of LPS control; as in Figure 6g) in human glomeruli ex vivo, BMI values of the study participants, and baseline expression levels of inflammation markers in glomeruli studied by immunohistochemistry (IHC). ***p* < 0.05** and ****p* < 0.01**. ARon, AdipoRon

		BMI	TLR4 (IHC)	p-NFκB (IHC)	CD68 (IHC)	IL-1β (ARon)	IL-6 (ARon)	IL-8 (ARon)	IL-10 (ARon)	IL-18 (ARon)	TNFα (ARon)
**BMI**	*r*	1									
	p										
**TLR4 (IHC)**	*r*	-0.380	1								
	p	0.314									
**p-NFκB (IHC)**	*r*	-0.225	**0.690***	1							
	p	0.560	**0.040**								
**CD68 (IHC)**	*r*	-0.457	0.389	0.627	1						
	p	0.216	0.300	0.071							
**IL-1β (ARon)**	*r*	0.292	-0.522	-0.407	-0.054	1					
	p	0.446	0.149	0.277	0.890						
**IL-6 (ARon)**	*r*	0.068	**-0.751***	-0.335	-0.039	**0.771***	1				
	p	0.863	**0.020**	0.378	0.920	**0.015**					
**IL-8 (ARon)**	*r*	0.099	-0.424	0.120	0.289	**0.758***	**0.824****	1			
	p	0.800	0.256	0.759	0.451	**0.018**	**0.006**				
**IL-10 (ARon)**	*r*	0.245	-0.403	-0.007	0.225	**0.823****	**0.835**	**0.872****	1		
	p	0.526	0.282	0.986	0.561	**0.006**	**0.005**	**0.002**			
**IL-18 (ARon)**	*r*	0.415	-0.347	-0.326	-0.368	**0.781***	0.449	0.530	0.501	1	
	p	0.267	0.360	0.392	0.330	**0.013**	0.225	0.142	0.170		
**TNFα (ARon)**	*r*	0.627	-0.665	-0.474	-0.114	**0.854****	0.652	0.597	**0.712***	0.576	1
	p	0.070	0.051	0.769	0.769	**0.003**	0.057	0.090	**0.031**	0.105	

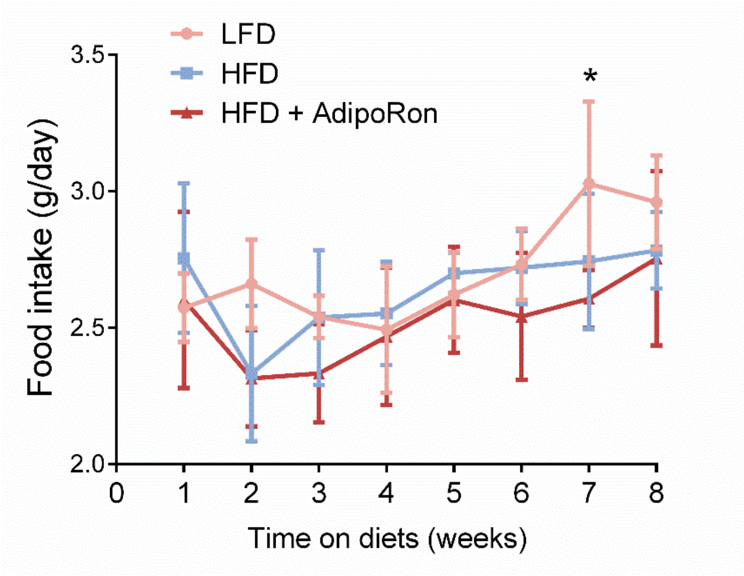

ESM Figure 1. Food intake of the mice. The weekly food consumption per cage was normalised to the number of mice (2) per cage. Data were assessed by one-way ANOVA at each time point with a subsequent Bonferroni or Tamhane's post hoc test depending on the homogeneity of variances. LFD, low-fat diet; HFD, high-fat diet. **p* < 0.05 HFD-AdipoRon versus LFD. LFD, low fat diet; HFD, high fat diet

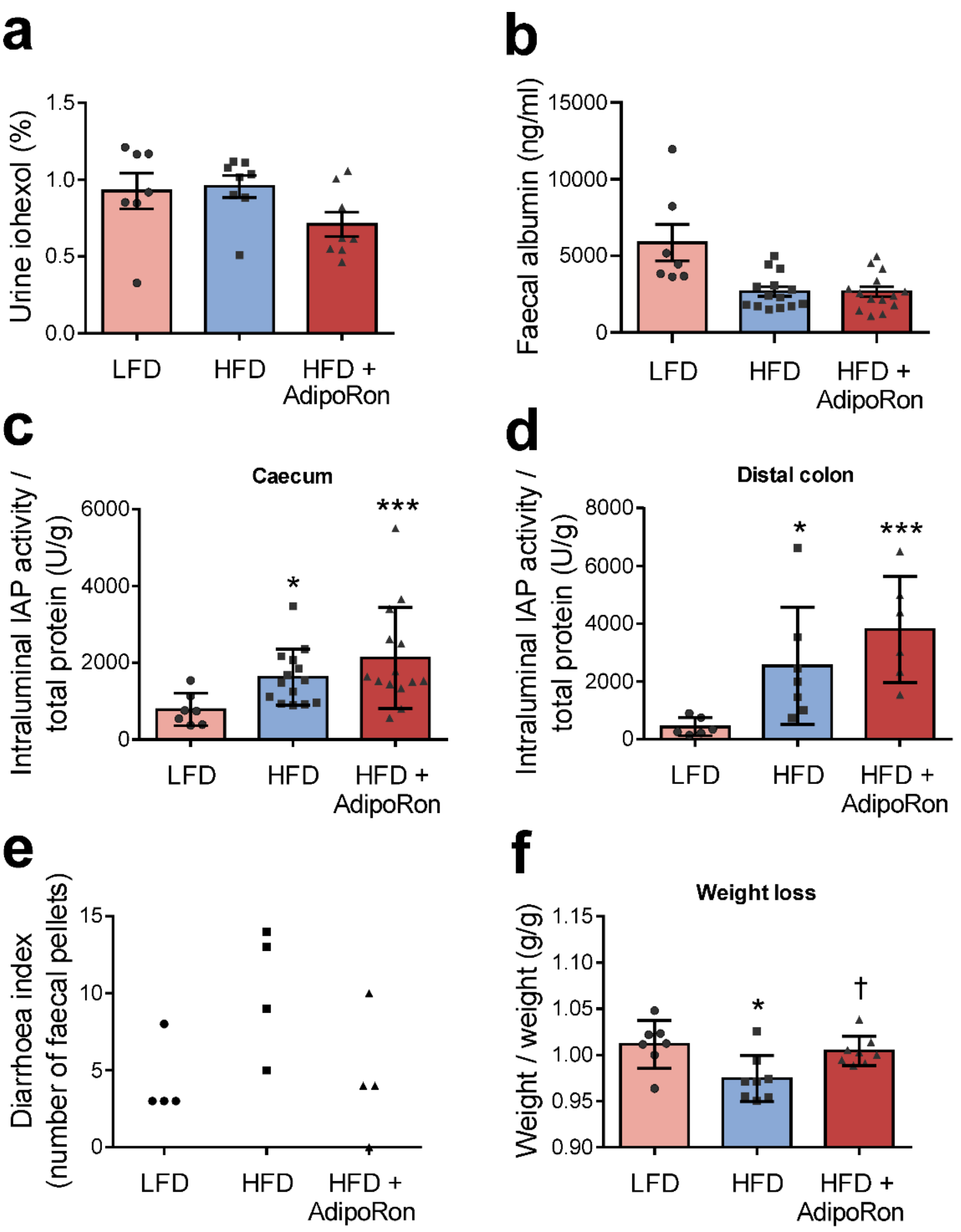

ESM Figure 2. The effects of AdipoRon administration on the intestine of HFD-fed mice. (**a**, **b**) Intestinal permeability measured by 24 h excretion of iohexol to urine (**a**) and the faecal levels of albumin (**b**). For iohexol: LFD, *n*=7; HFD, *n*=8; HFD-AdipoRon, *n*=8. For albumin: LFD, *n*=7; HFD, *n*=14; HFD-AdipoRon, *n*=14. (**c**, **d**) The activity of intestinal alkaline phosphatase (IAP) in the intraluminal contents of caecum (**c**) and distal colon (**d**). For caecum: LFD, *n*=7; HFD, *n*=14; HFD-AdipoRon, *n*=14. For distal colon: LFD, *n*=6; HFD, *n*=7; HFD-AdipoRon, *n*=6. (**e**) Diarrhoea index represented as the number of faecal pellets adhered to the bottom grid of the metabolic cage after 24 h administration of iohexol (*n*=4 in each group). (**f**) Relative body weight loss in response to the iohexol experiment represented as the ratio of the body weight 4 days before and 4 days after iohexol administration. LFD, *n*=7; HFD, *n*=8; HFD-AdipoRon, *n*=8. Data throughout were assessed by one-way ANOVA (normal distribution) or Kruskal Wallis test (non-normal distribution) with a subsequent Bonferroni or Tamhane's post hoc test depending on the homogeneity of variances. **p* < 0.05 and ****p* < 0.001 versus LFD group. †*p* < 0.05 versus HFD group. LFD, low fat diet; HFD, high fat diet

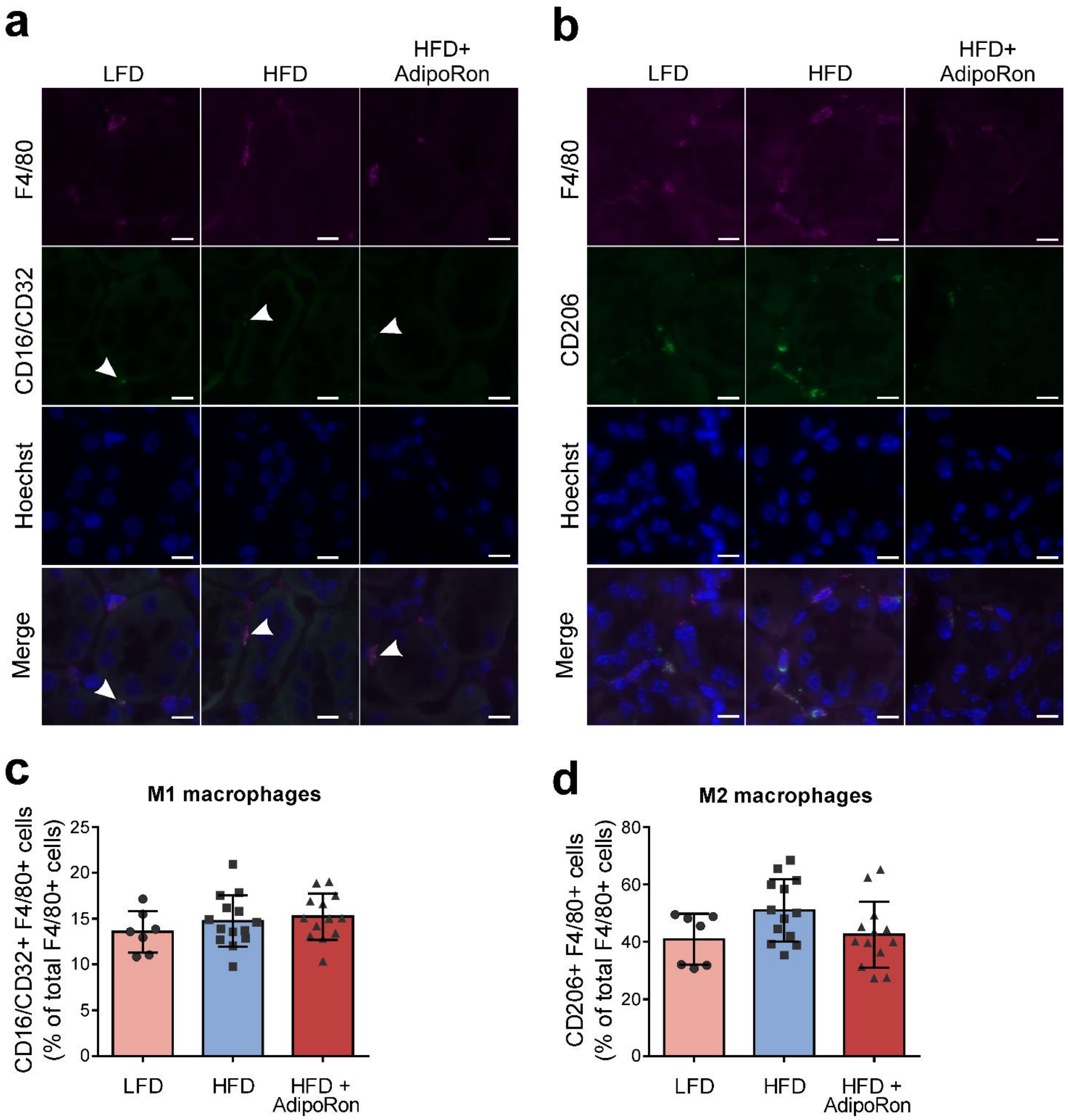

ESM Figure 3. The effects of AdipoRon administration on macrophage polarization in the kidneys of HFD-fed mice. (**a**, **b**) Representative images of immunofluorescence stainings on frozen renal sections for F4/80 (red) and CD16/CD32 (green) (**a**) and F4/80 (red) and CD206 (green) (**b**). Hoechst 33342-stained nuclei are shown in blue. Arrowheads indicate double-positive (F4/80+ CD16/CD32+ or F4/80+ CD206+) cells. Scale bar, 10 µm. (**c**) Quantification of the percentage of CD16/CD32+ F4/80+ macrophages (M1 phenotype) and (**d**) the percentage of CD206+ F4/80+ macrophages (M2 phenotype) among total F4/80+ macrophages as stained in (**a**, **b**). For M1 macrophages: LFD, *n*=7; HFD, *n*=14; HFD-AdipoRon, *n*=13. For M2 macrophages: LFD, *n*=7; HFD, *n*=13; HFD-AdipoRon, *n*=13. Data throughout were assessed by one-way ANOVA with a subsequent Bonferroni post hoc test. LFD, low fat diet; HFD, high fat diet

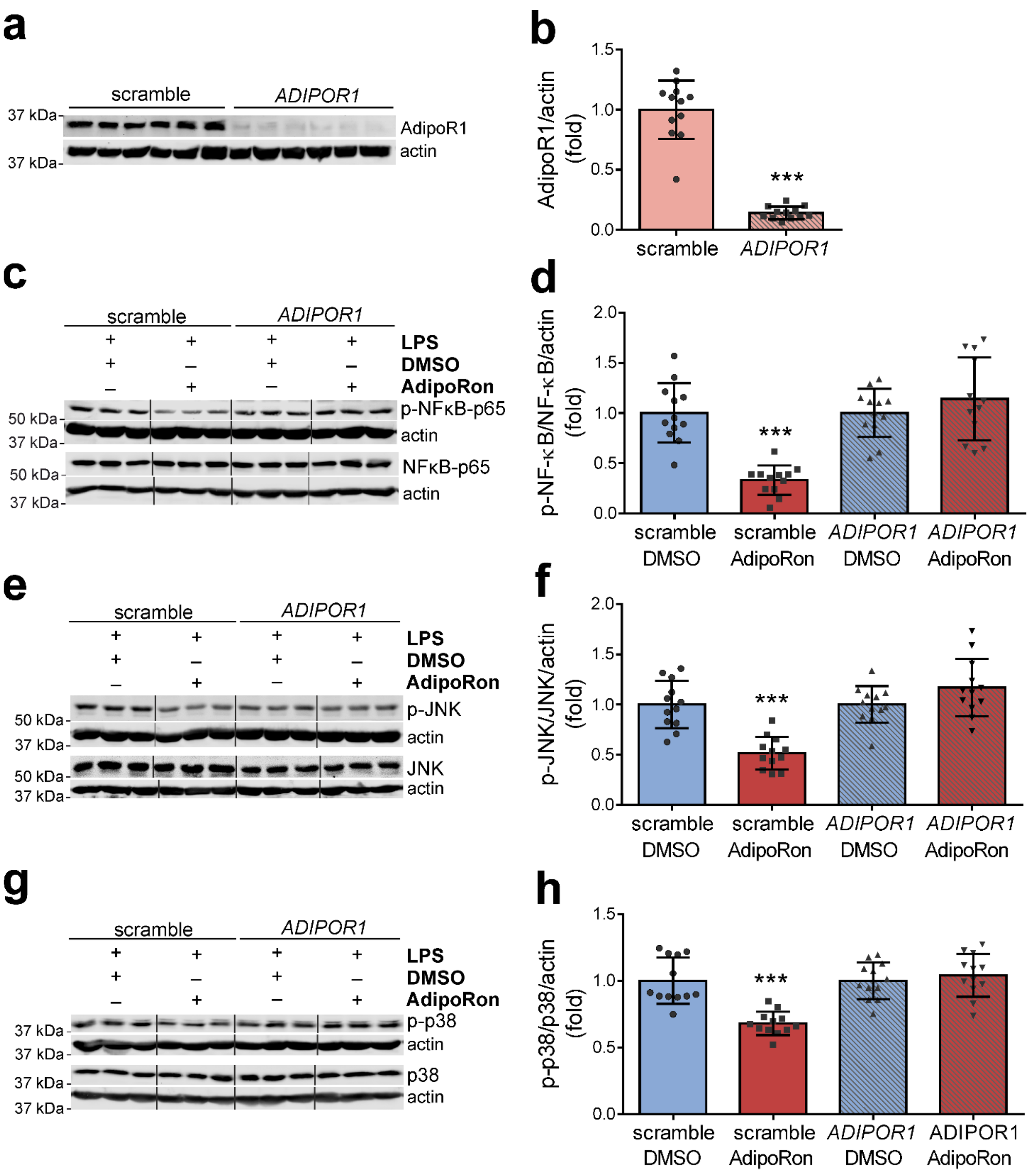

ESM Figure 4. The effect of AdipoRon on inflammatory signalling pathways in human podocytes with stable AdipoR1 knockdown. (**a**, **b**) Validation of the stable shRNA-mediated AdipoR1 knockdown in cultured human podocytes. ***p < 0.001 versus scramble. (**c**–**h**) Representative immunoblots of lysates prepared from control (scramble shRNA) and *ADIPOR1* shRNA human podocytes treated with AdipoRon or DMSO and stimulated with LPS for 1 h (**c**, **e**, **g**), and the expression levels of phospho-NF-κB-p65/NF-κB-p65 (**d**), phospho-JNK (p54 isoform)/JNK (**f**) and phospho-p38-MAPK/p38-MAPK (**h**) normalised to actin quantified from four independent experiments as in (**c**, **e**, **g**). Results are normalised to DMSO-treated control. Data throughout were assessed by unpaired two-tailed Student's t test. ***p < 0.001 versus DMSO

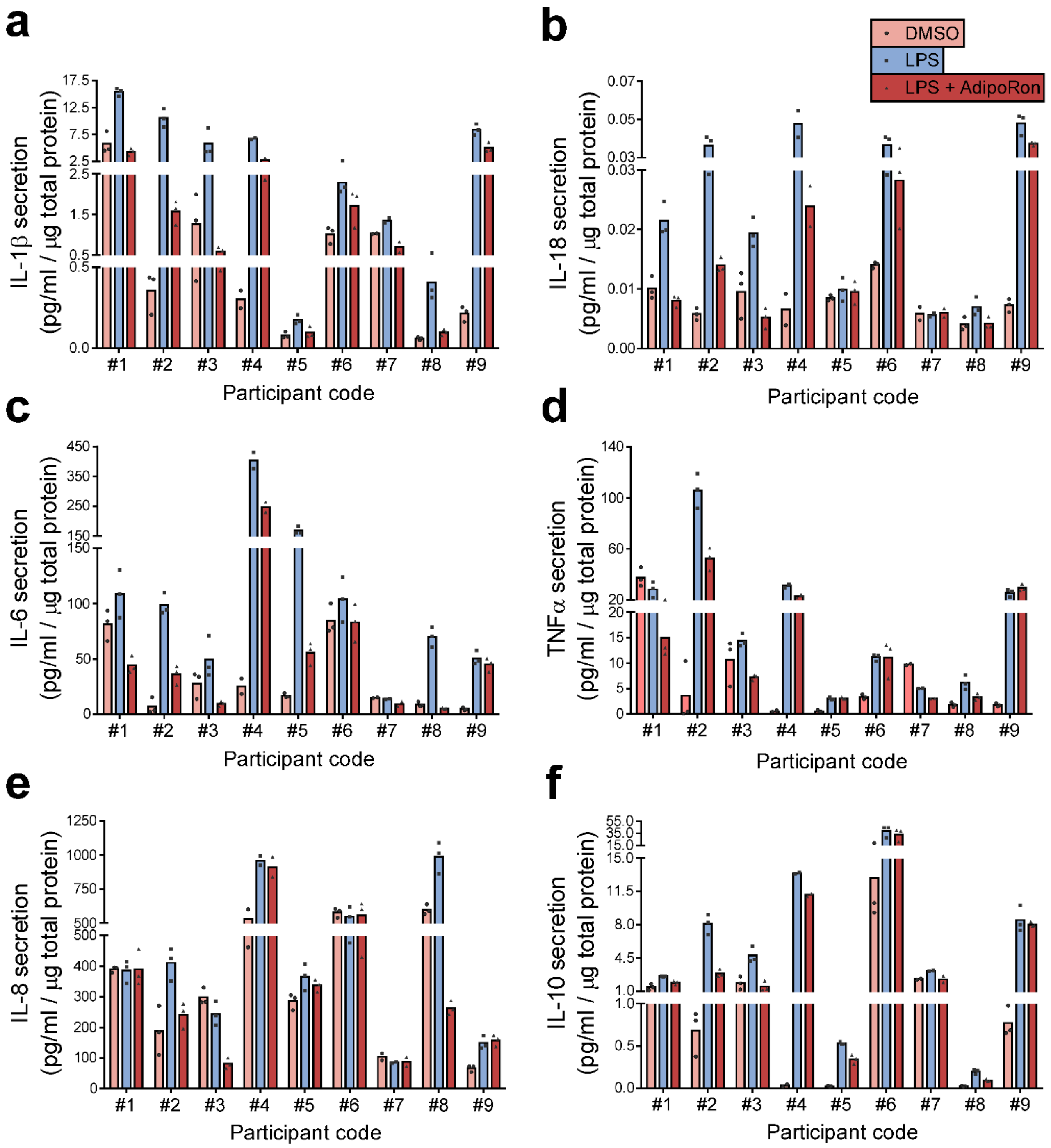

ESM Figure 5. The LPS-induced cytokine secretion with and without AdipoRon in human glomeruli between the study participants. (**a**–**f**) The concentrations (pg/ml) of IL-1β (**a**), IL-18 (**b**), IL-6 (**c**), TNFα (**d**), IL-8 (**e**) and IL-10 (**f**) secreted into the culture media normalised to the total protein content (µg) of the corresponding glomerular lysate of DMSO-, LPS- or LPS-AdipoRon-treated (24 h) human glomeruli (n=3 wells per treatment except for #4 and #7 where n=2). Bar graphs present the mean value of the replicates. The characteristics of the study participants are described in ESM Table 2.

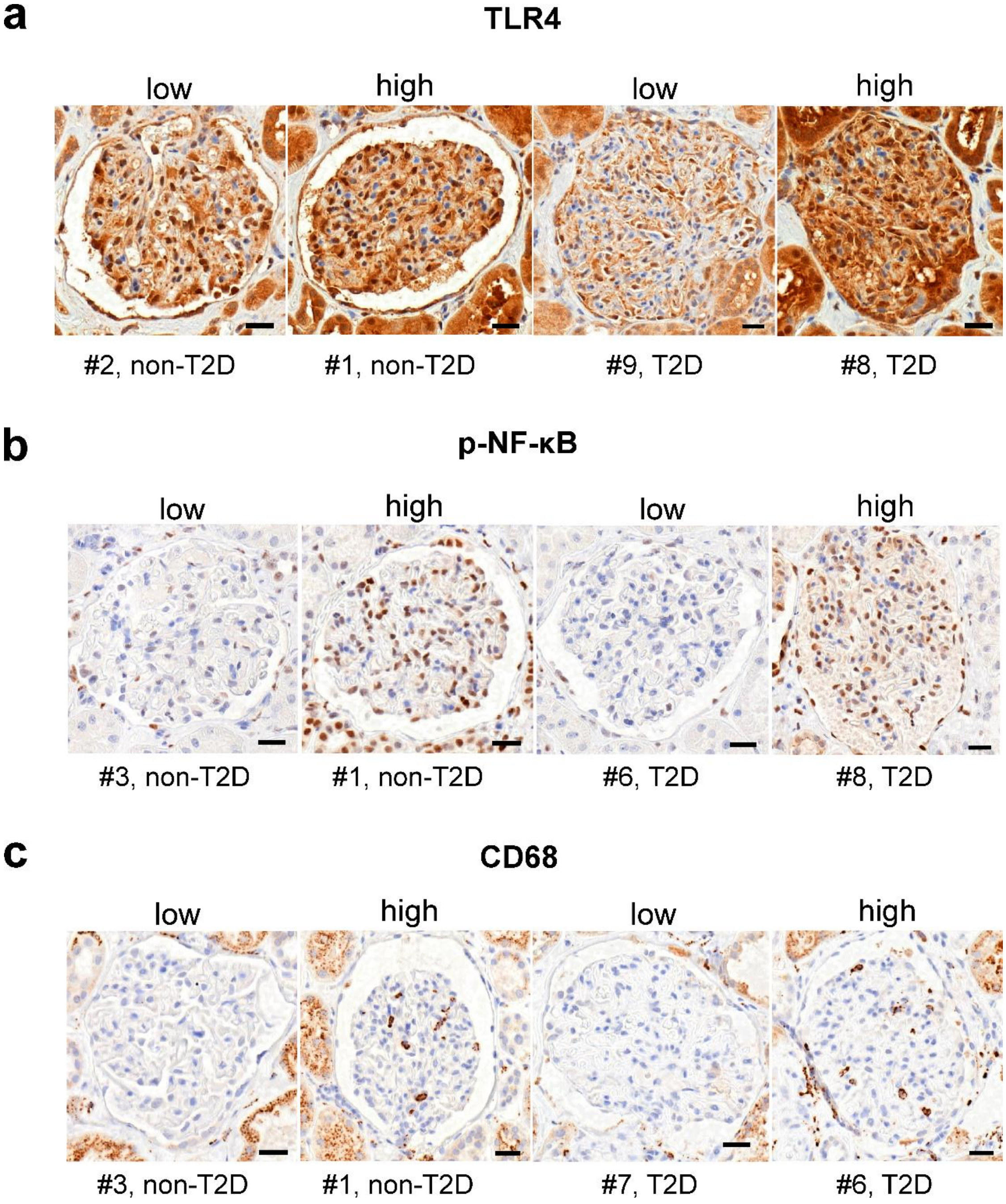

ESM Figure 6. Immunohistochemical stainings for TLR4, phospho-NF-κB and CD68 on human kidneys used in ex vivo experiments. (**a**–**c**) Representative immunohistochemical stainings showing low and high glomerular expression level of TLR4 (**a**), phospho-NF-κB-p65 (**b**), and CD68 (**c**) in study participants with and without type 2 diabetes (T2D). ID number (#) of the study participants as presented in ESM Table 2 is indicated below the image. Scale bar, 25 µm

References

[1] Okada-Iwabu M, Yamauchi T, Iwabu M et al (2013) A small-molecule AdipoR agonist for type 2 diabetes and short life in obesity. Nature 503(7477):493-499. https://doi.org/10.1038/nature12656

[2] Kim Y, Lim JH, Kim MY et al (2018) The Adiponectin Receptor Agonist AdipoRon Ameliorates Diabetic Nephropathy in a Model of Type 2 Diabetes. J Am Soc Nephrol 29(4):1108-1127. https://doi.org/10.1681/ASN.2017060627

[3] Hyvonen ME, Dumont V, Tienari J et al (2015) Early-Onset Diabetic E1-DN Mice Develop Albuminuria and Glomerular Injury Typical of Diabetic Nephropathy. Biomed Res Int. 2015:102969. https://doi.org/10.1155/2015/102969

[4] Forsgård RA, Korpela R, Holma R et al (2016) Intestinal permeability to iohexol as an in vivo marker of chemotherapy-induced gastrointestinal toxicity in Sprague-Dawley rats. Cancer Chemother Pharmacol 78(4):863-874. https://doi.org/10.1007/s00280-016-3150-3

[5] Lassenius MI, Fogarty CL, Blaut M et al (2017) Intestinal alkaline phosphatase at the crossroad of intestinal health and disease – a putative role in type 1 diabetes. J Intern Med 281(6):586-600. https://doi.org/10.1111/joim.12607

[6] McQuin C, Goodman A, Chernyshev V et al (2018) CellProfiler 3.0: Next-generation image processing for biology. PLoS Biol 16(7):e2005970. https://doi.org/10.1371/journal.pbio.2005970

[7] Saleem MA, O'Hare MJ, Reiser J et al (2002) A Conditionally Immortalized Human Podocyte Cell Line Demonstrating Nephrin and Podocin Expression. J Am Soc Nephrol 13(3):630-638.

[8] Datta N, Lindfors S, Miura N, Saleem MA, Lehtonen S (2016) Overexpression of transcription factor FOXC2 in cultured human podocytes upregulates injury markers and increases motility. Exp Cell Res 340(1):32-42. https://doi.org/10.1016/j.yexcr.2015.10.035

[9] Saurus P, Kuusela S, Lehtonen E et al (2015) Podocyte apoptosis is prevented by blocking the Toll-like receptor pathway. Cell death & disease 6(5):e1752. https://doi.org/10.1038/cddis.2015.125

[10] Sluch VM, Banks A, Li H et al (2018) ADIPOR1 is essential for vision and its RPE expression is lost in the Mfrprd6 mouse. Scientific Reports 8(1):14339. https://doi.org/10.1038/s41598-018-32579-9
